# Environment random interaction of rime optimization with Nelder-Mead simplex for parameter estimation of photovoltaic models

**DOI:** 10.1038/s41598-024-65292-x

**Published:** 2024-07-08

**Authors:** Jinge Shi, Yi Chen, Ali Asghar Heidari, Zhennao Cai, Huiling Chen, Yipeng Chen, Guoxi Liang

**Affiliations:** 1 Institute of Big Data and Information Technology, Wenzhou University, Wenzhou, 325035 China; 2 School of Surveying and Geospatial Engineering, College of Engineering, University of Tehran, Tehran, Iran; 3 Center of AI Technology Application R&D, Wenzhou Polytechnic, Wenzhou, 325035 China; 4 Department of Artificial Intelligence, Wenzhou Polytechnic, Wenzhou, 325035 China

**Keywords:** Rime optimization algorithm, Nelder-Mead, Solar energy, Photovoltaic models, Global optimization, Computational science, Computer science

## Abstract

As countries attach importance to environmental protection, clean energy has become a hot topic. Among them, solar energy, as one of the efficient and easily accessible clean energy sources, has received widespread attention. An essential component in converting solar energy into electricity are solar cells. However, a major optimization difficulty remains in precisely and effectively calculating the parameters of photovoltaic (PV) models. In this regard, this study introduces an improved rime optimization algorithm (RIME), namely ERINMRIME, which integrates the Nelder-Mead simplex (NMs) with the environment random interaction (ERI) strategy. In the later phases of ERINMRIME, the ERI strategy serves as a complementary mechanism for augmenting the solution space exploration ability of the agent. By facilitating external interactions, this method improves the algorithm’s efficacy in conducting a global search by keeping it from becoming stuck in local optima. Moreover, by incorporating NMs, ERINMRIME enhances its ability to do local searches, leading to improved space exploration. To evaluate ERINMRIME's optimization performance on PV models, this study conducted experiments on four different models: the single diode model (SDM), the double diode model (DDM), the three-diode model (TDM), and the photovoltaic (PV) module model. The experimental results show that ERINMRIME reduces root mean square error for SDM, DDM, TDM, and PV module models by 46.23%, 59.32%, 61.49%, and 23.95%, respectively, compared with the original RIME. Furthermore, this study compared ERINMRIME with nine improved classical algorithms. The results show that ERINMRIME is a remarkable competitor. Ultimately, this study evaluated the performance of ERINMRIME across three distinct commercial PV models, while considering varying irradiation and temperature conditions. The performance of ERINMRIME is superior to existing similar algorithms in different irradiation and temperature conditions. Therefore, ERINMRIME is an algorithm with great potential in identifying and recognizing unknown parameters of PV models.

## Introduction

With the development of the times, energy conservation and environmental protection have become the theme of this twenty-first century^1^. Energy is vital in economic and social development^2,3^. How to achieve both environmental protection and the supply of energy^4^ to society is a major social issue. The emergence of environmentally friendly energy sources^5^ other than fossil fuel energy has solved this major problem. Among them, The acquisition of solar energy is well-known and widely used due to its small regional restrictions and simple acquisition methods^6^. An important renewable energy source in the modern era is solar energy, which can be harnessed and transformed into electricity, which is a vital energy source in the modern era^7^. Therefore, solar energy is an appropriate renewable energy source to achieve sustainable economic development as an alternative to fossil fuel energy^8^. Similar to other renewable energies, solar energy requires equipment to convert it into electricity to meet the needs of society^9^. The photovoltaic (PV) models^10^ came into being. Scientists applied PV technology to solar energy systems to reduce the loss rate in the energy conversion process and improve its energy conversion efficiency^11^. Nevertheless, the design of PV models is often susceptible to external disturbances, and the impact of adverse weather conditions can significantly affect the accuracy of the model parameters^12^. Moreover, as time passes, various components within the model may be corroded and eroded by air, which can produce inaccurate results^13^. Therefore, managing PV models equipment becomes particularly important^14^. Accurate predictive simulation of PV models can help management quickly determine equipment status and carry out maintenance and replacement. Therefore, the relevant parameters for constructing the model must be accurately predicted, and the most important is the prediction of unknown parameters^15^. The single diode model (SDM)^16^, double diode model (DDM)^17^, three diode model (TDM)^18^, and PV module model^19^ equations are all implicit transcendental equations. Accurately predicting the relevant parameters for these models is extremely complex^20^.

Predicting these parameters has become the focus of many experts and scholars. Two general categories can be used to evaluate parameters in PV models: deterministic algorithms and stochastic algorithms^21^. Deterministic algorithms mainly solve calculations based on statistical or mathematical methods, such as analytical methods, numerical calculation methods, and iterative curve fitting. However, because extracting parameters for PV models is complicated, deterministic methods are not very effective for complex uncertain problems that are non-convex, and non-differentiable^22^. Various limiting factors often constrain the extraction of PV model parameters^23^. So, in such a context, optimization and stochastic algorithms have been appeared in recent years^24^. Among which meta-heuristic algorithms (MAs) have drawn a lot of interest lately because of their straightforward, efficient, and adaptable methods^25^. MAs are mainly created based on inspirations from the habits of social groups or animals^26^, as well as physical phenomena or mathematical states. They simplify complex problems by iterating based on the function of specific mathematical formulas and eventually approach the global optimal solution.

Currently, MAs can be divided roughly four main groups^27^: evolutionary algorithms, algorithms inspired by animal and plant populations, algorithms based on physical or mathematical principles, and algorithms inspired by human activities. Inspired by the concepts of biological evolution, evolutionary algorithms mimic the natural population’s evolutionary process^28^. These algorithms employ genetic variation, mutation, and selection mechanisms to search for optimal solutions. The most reputable, applied evolutionary algorithms include Genetic Algorithms (GA)^29^, Simulated Annealing (SA)^30^, Genetic Programming (GP)^31^, and Differential Evolution (DE)^32^. Some algorithms draw insights from the collective intelligence. Among them, the most typical algorithms include Ant Colony Optimization (ACO)^33^, Harris Hawks Optimization (HHO)^34^, Grey Wolf Optimization (GWO)^35^, Hunger Games Search (HGS)^36^, Whale Optimization Algorithm (WOA)^37^, Parrot Optimizer (PO)^38^, Colony Predation Algorithm (CPA)^39^, Slime Mold Algorithm (SMA)^40^, and others. Algorithms based on physical or mathematical principles are a category of optimization algorithms that simulate physical phenomena or mathematical principles to search for optimal solutions. Representative algorithms include Simulated Annealing (SA)^41^, Thermal Exchange Optimization (TEO)^42^, Multi-verse Optimizer (MVO)^43^, Rime Optimization Algorithm (RIME)^44^, Runge Kutta Optimizer (RUN)^45^, Weighted Mean Of Vectors (INFO)^46^, and others. Algorithms based on human activity behavior are a category of optimization algorithms that solve problems by simulating human behavior and learning processes^47^. Typical algorithms in this category include Teaching–Learning-based Optimization (TLBO)^48^, Volleyball Premier League (VPL)^49^, Liver Cancer Algorithm (LCA)^50^, and Gaining Sharing Knowledge (GSK)^51^.

At present, evolutionary methods and MAs have been widely used in topology optimization^52^, image segmentation^53^, feature selection^54^, engineering design^55^, dispatch problem^56^, intrusion detection^57^, and many other fields. For these complex optimization problems, MAs have shown remarkable results in their applications. For instance, Li et al. suggested a hybrid method based on PSO and GWO to tackle net electricity consumption prediction with an efficient performance^58^. Xu *et al*. proposed incorporating chaotic mutation into Moth–Flame Optimization (MFO) to improve the algorithm's capacity to avoid local optima^59^. It was applied to predicting financial pressure scenarios. Hu et al. proposed a dispersion foraging SMA^60^. Gupta proposed an improved GWO based on a random walk^61^. This improved version has demonstrated proficiency in solving both continuous optimization problems. Hussain *et al*. proposed a combination of the Sine–Cosine Algorithm (SCA) and the HHO^62^. It was conducted on feature selection problems in both low-dimensional and high-dimensional spaces. There has been a proliferation of outstanding MAs in the realm of parameter evaluation for PV models. For example, Peng et al. introduced the Nelder-Mead simplex (NMs) in conjunction with an improved WOA^63^. Weng *et al*. proposed the integration of the Backtracking Search Optimization Algorithm (BSA) and TLBO^64^. It has been used in the energy PV models. Chen *et al*. proposed a diversification-enhanced HHO^40^ to effectively estimate the unknown parameters of PV models.

RIME is an innovative optimization algorithm inspired by physical phenomena, precisely mimicking the formation of both soft and hard rime ice. RIME has garnered significant attention and recognition from many scholars due to its advantages, such as a small number of control parameters and remarkable optimization ability. However, it is essential to acknowledge that while the algorithm has demonstrated potential in various optimization tasks, its applicability to identifying unknown parameters of PV models remains constrained, as demonstrated by the "no free lunch theorem"^65^. One of the algorithm’s shortcomings is its insufficient global exploration ability, which could result getting stuck local optima. Therefore, this work has extended this algorithm to improve its overall performance for parameter identification of PV models. This study introduces an improved optimization algorithm named ERINMRIME, built upon the foundation of RIME. ERINMRIME incorporates the environment random interaction (ERI) strategy and the NMs mechanism to enhance the accuracy of evaluating PV models.

Since the rime particles (the individuals within the population) generated by RIME do not exist stably in the environment. The particles constantly change with the environment. The original RIME lacks the interaction of particles with the environment in the later iteration phase. In other words, these particles among RIME are not changed due to the changes in wind speed, temperature, and other external conditions in the living environment at the later stage of the algorithm. This study introduces an ERI strategy to address this issue and enhance the interaction capability between rime particles and the environment. Integrating the ERI strategy into ERINMRIME significantly broadens the algorithm's global search space, reducing the possibility of being stuck in local optima. Including the NMs mechanism in ERINMRIME significantly enhances the algorithm's local search capability, enabling it to efficiently optimize inferior solutions and improve the overall quality of solutions. The mechanism thoroughly explores the valuable solution space, improving performance in solving complex optimization problems. Fusing the two mechanisms in ERINMRIME ultimately results in a balanced approach, achieving local and global equilibrium states. To confirm that ERINMRIME is a reliable tool for estimating PV models unknown parameters, this research conducted a comprehensive comparison with other widely used advanced algorithms for PV optimization problems. The extensive experimental results demonstrate that ERINMRIME exhibits distinctive and dominant competitiveness in addressing PV optimization problems, underscoring its superiority and efficacy in this particular domain.

Generally speaking, the following are the study’s contributions:

- To achieve more efficient and accurate extraction of unknown parameters in PV models, this study proposed an enhanced version of RIME, referred to as ERINMRIME.
- This study introduced an ERI strategy to further interaction between rime particles and the environment in the later iteration stage. This tactic successfully reduced the algorithm’s propensity to become trapped in local optima in local optima.
- This study introduced the NMs mechanism, which enhanced the quality of the solutions and improved the algorithm's capability for local exploitation.
- ERINMRIME was subjected to rigorous evaluation against previous advanced algorithms in PV models, affirming its reliability and value in accurately assessing PV problems.
- The practical efficacy of ERINMRIME in parameter extraction from commercial PV models was assessed through testing in diverse and complex environments.

The remaining organizational structure of this paper is as follows: Section "Problem formulations" introduces the relevant PV models. Section "The proposed algorithm" comprehensively describes the proposed algorithm, detailing its key components and mechanisms. Section "Experiments and analysis for benchmark functions" verifies the effectiveness of ERINMRIME by practical experiments on IEEE CEC benchmark functions. Section "Experimental and analysis for PV models" verifies the effectiveness of ERINMRIME by practical experiments on PV models. Section "Discussion on the results" evaluates and summarizes the experimental results. Section "Conclusions and future directions" examines the potential applications of this research and provides an overview of the entire work.

## Problem formulations

In the quest for improved accuracy and efficiency in assessing the I-V characteristics of PV models, researchers have dedicated their efforts to developing various models for PV systems. Within the domain of PV modeling, numerous models have been put forward. This study presents three general PV models, namely SDM, DDM, and TDM, which can be applied to all types of PV generators, including both individual cells and modules. Additionally, this section introduces the PV component models and the objective functions involved in this research.

### General model

#### SDM

**Figure 1:**
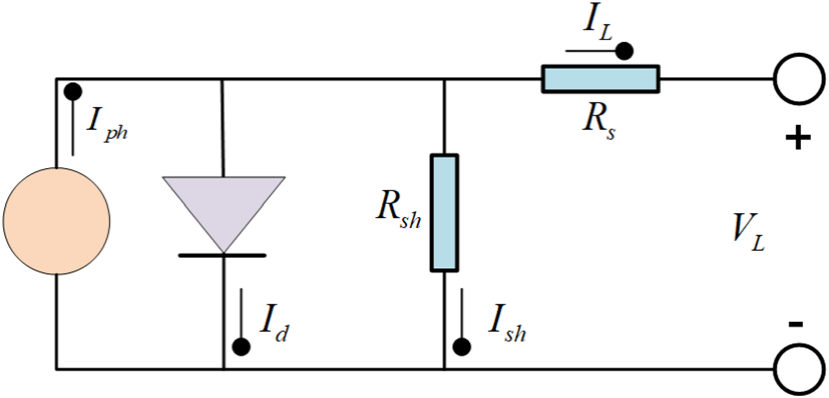
SDM.

When evaluating energy cases, the utilization of a precise and effective model is principal. To this end, SDM was developed to depict the PV landscape of solar energy accurately. The equivalent circuit diagram of the SDM is illustrated in Fig. 1. The model encompasses a current source operating in parallel with a diode, a shunt resistor representing the leakage current, and a series resistor accounting for the losses associated with load current^66^. The output current is calculated by Eq. (1).

1 $$I_{L} = I_{ph} - I_{d} - I_{sh}$$

where $I_{L}$ represents the output current of the SDM. $I_{ph}$ is the current generated by the light. $I_{d}$ represents the current through the diode, and $I_{sh}$ represents the current through the shunt resistor. $I_{d}$ is calculated as shown in Eq. (2).

2 $$I_{d} = I_{sd} \times \left[ {\exp \left( {\frac{{q \times (V_{L} + R_{s} \times I_{L} )}}{n \times k \times T}} \right) - 1} \right]$$

where $I_{sd}$ represents the reverse saturation current. $q$ is the elementary charge ($1.60217646 \times 10^{ - 19} C$). $V_{L}$ is the output voltage. $R_{s}$ represents the series resistance. $n$ is the ideal factor of the diode. $k$ is the Boltzmann constant ($1.3806503 \, \times \, 10^{23} J/K$), and *T* is the temperature in Kelvin. The current $I_{sh}$ through the shunt resistor can be obtained by Eq. (3).

3 $$I_{sh} = \frac{{V_{L} + R_{s} \times I_{L} }}{{R_{sh} }}$$

where $R_{sh}$ represents shunt resistance, and Eq. (1) is further expressed by Eq. (4).

4 $$I_{L} = I_{ph} - I_{sd} \times \left[ {\exp \left( {\frac{{q \times (V_{L} + R_{s} \times I_{L} )}}{n \times k \times T}} \right) - 1} \right] - \frac{{V_{L} + R_{s} \times I_{L} }}{{R_{sh} }}$$

#### DDM

Due to the drawbacks of the SDM, such as neglecting the temperature effect, lacking accurate nonlinear description, and limited parameterization flexibility, this study introduces the DDM as the benchmark model to enhance the universality and comparability of research results, facilitating easier comparison and evaluation of different algorithms' performance.

**Figure 2:**
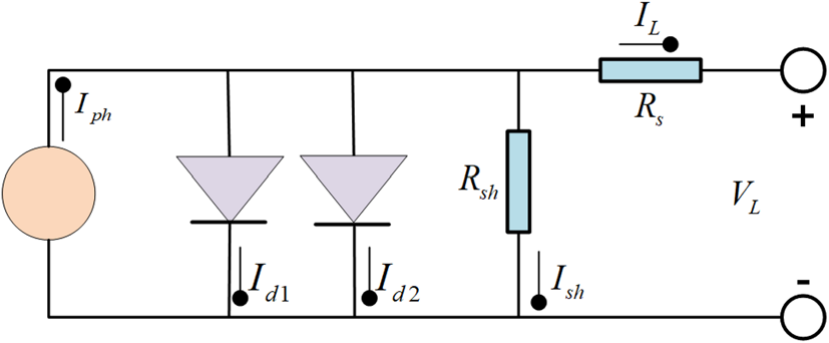
DDM.

The DDM incorporates a diode and a photogenerated current source in series to simulate the compound current^67^. The equivalent circuit diagram of the DDM is depicted in Fig. 2. The final current generated by DDM is shown in Eq. (5).

5 $$I_{L} = I_{ph} - I_{d1} - I_{d2} - I_{sh}$$

According to Eq. (4), we can write Eq. (5) as Eq. (6).

6 $$I_{L} = I_{ph} - I_{sd1} \times \left[ {\exp \left( {\frac{{q \times (V_{L} + R_{s} \times I_{L} )}}{{n_{1} \times k \times T}}} \right) - 1} \right] - I_{sd2} \times \left[ {\exp \left( {\frac{{q \times (V_{L} + R_{s} \times I_{L} )}}{{n_{2} \times k \times T}}} \right) - 1} \right] - \frac{{V_{L} + R_{s} \times I_{L} }}{{R_{sh} }}$$

where $n_{1}$ and $n_{2}$ represent the ideal coefficient of diffusion and composite diode. $I_{sd1}$ and $I_{sd2}$ represent the diode diffusion and saturation current, and other parameters are the same as the above formula, respectively.

#### TDM

While the DDM offers improved accuracy, it is still deemed inadequate in accurately resolving large-scale and intricate engineering challenges. This study introduces the more precise TDM as a benchmark model to further improve the accuracy and comparability of research results. By incorporating the TDM, algorithm performance and robustness can be better evaluated, especially under complex operating conditions. This contributes to advancing research and development in photovoltaic systems and facilitates comparisons and evaluations among different algorithms.

**Figure 3:**
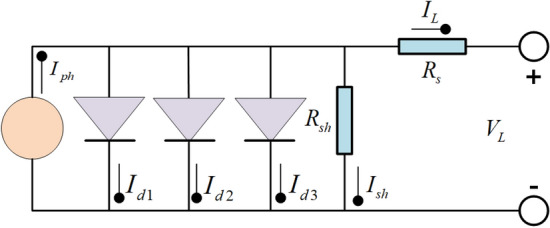
TDM.

The TDM incorporates an additional diode based on the DDM^68^. The equivalent circuit diagram of the TDM is illustrated in Fig. 3. The ultimate output current of the TDM can be obtained by Eq. (7).

7 $$\begin{aligned} I_{L} = & I_{ph} - I_{sd1} \times \left[ {\exp \left( {\frac{{q \times (V_{L} + R_{s} \times I_{L} )}}{{n_{1} \times k \times T}}} \right) - 1} \right] - I_{sd2} \times \left[ {\exp \left( {\frac{{q \times (V_{L} + R_{s} \times I_{L} )}}{{n_{2} \times k \times T}}} \right) - 1} \right] \\ & \quad - I_{sd3} \times \left[ {\exp \left( {\frac{{q \times (V_{L} + R_{s} \times I_{L} )}}{{n_{3} \times k \times T}}} \right) - 1} \right] - \frac{{V_{L} + R_{s} \times I_{L} }}{{R_{sh} }} \\ \end{aligned}$$

where $I_{sd3}$ and $n_{3}$ represent the current flowing through the third diode and its ideal factor.

### PV module model

**Figure 4:**
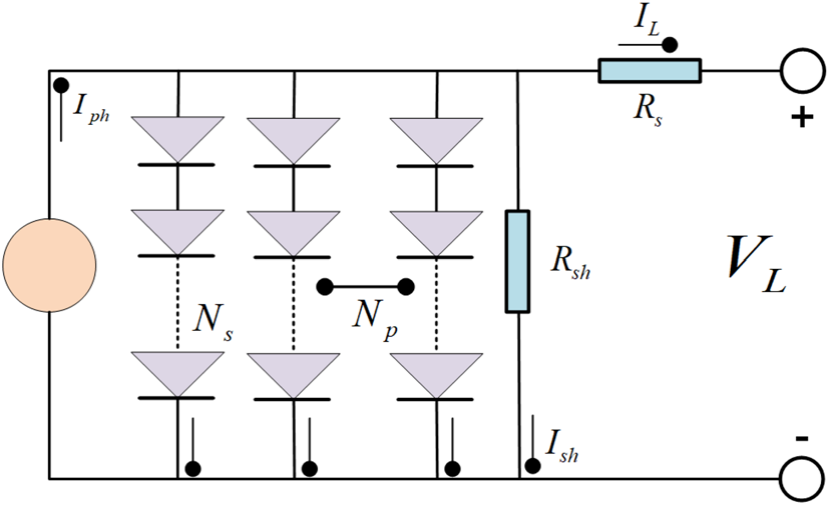
PV module model.

The PV module model typically consists of multiple models interconnected in a series or parallel arrangement^69^. The equivalent circuit of the PV module model is presented in Fig. 4. The output current of the PV module model, based on the SDM and DDM, can be mathematically expressed by Eq. (8) and Eq. (9), respectively.

8 $$I_{L} = I_{ph} N_{p} - I_{sd} \times N_{p} \times \left[ {\exp \left( {\frac{{q \times (\frac{{V_{L} }}{{N_{s} }} + N_{s} \times R_{s} \times \frac{{I_{L} }}{{N_{p} }})}}{{n \times k \times N_{s} \times T}}} \right) - 1} \right] - \frac{{\frac{{V_{L} }}{{N_{s} }} + N_{s} \times R_{s} \times \frac{{I_{L} }}{{N_{p} }}}}{{\frac{{R_{sh} \times N_{s} }}{{N_{p} }}}}$$

9 $$\begin{aligned} I_{L} = & I_{ph} N_{p} - I_{sd1} \times N_{p} \times \left[ {\exp \left( {\frac{{q \times (\frac{{V_{L} }}{{N_{s} }} + N_{s} \times R_{s} \times \frac{{I_{L} }}{{N_{p} }})}}{{n_{1} \times k \times T \times N_{s} }}} \right) - 1} \right] \\ & \quad - I_{sd2} \times N_{p} \times \left[ {\exp \left( {\frac{{q \times (\frac{{V_{L} }}{{N_{s} }} + N_{s} \times R_{s} \times \frac{{I_{L} }}{{N_{p} }})}}{{n_{2} \times k \times T \times N_{s} }}} \right) - 1} \right] \\ & \quad - \frac{{\frac{{V_{L} }}{{N_{s} }} + N_{s} \times R_{s} \times \frac{{I_{L} }}{{N_{p} }}}}{{\frac{{R_{sh} \times N_{s} }}{{N_{p} }}}} \\ \end{aligned}$$

where $N_{p}$ is the number of parallel solar cells. $N_{s}$ is the number of series solar cells.

### Objective function

To improve the fitting of the data of the $I_{L}$ and $V_{L}$ of PV models, this study adopts the Root Mean Square Error (*RMSE*) as the optimization objective to minimize the discrepancies between the measured data and the real values. A smaller *RMSE* indicates a better fit of the data. The specific formula for *RMSE* is as follows:

10 $$RMSE(X) = \sqrt {\frac{1}{N}\sum\limits_{i = 1}^{N} {f_{{\text{i}}}^{2} (V_{L} ,I_{L} ,X)} } ,i = 1,2,...,N;$$

where $I_{L}$ and $V_{L}$ are the output current and voltage of the model, respectively. *N* represents the amount of measured current data. *X* represents the solution vector containing unknown parameters.

**Equation **(11)**—Eq. **(13) represent the *RMSE* functions ($f$) for SDM, DDM, and TDM, respectively.

11 $$f(V_{L} ,I_{L} ,X) = I_{ph} - I_{L} - I_{sd} \times \left[ {\exp \left( {\frac{{q \times (V_{L} + R_{s} \times I_{L} )}}{n \times k \times T}} \right) - 1} \right] - \frac{{V_{L} + R_{s} \times I_{L} }}{{R_{sh} }}$$

12 $$\begin{aligned} f(V_{L} ,I_{L} ,X) = & I_{ph} - I_{L} - I_{sd1} \times \left[ {\exp \left( {\frac{{q \times (V_{L} + R_{s} \times I_{L} )}}{{n_{1} \times k \times T}}} \right) - 1} \right] \\ & \quad - \left[ {\exp \left( {\frac{{(R_{s} \times I_{L} + V_{L} )}}{{k \times T \times n_{2} }} \times q} \right) - 1} \right] \times I_{sd2} - \frac{{V_{L} + R_{s} \times I_{L} }}{{R_{sh} }} \\ \end{aligned}$$

13 $$\begin{aligned} f(V_{L} ,I_{L} ,X) = & I_{ph} - I_{sd1} \times \left[ {\exp \left( {\frac{{q \times (V_{L} + R_{s} \times I_{L} )}}{{n_{1} \times k \times T}}} \right) - 1} \right] - I_{sd2} \times \left[ {\exp \left( {\frac{{q \times (V_{L} + R_{s} \times I_{L} )}}{{n_{2} \times k \times T}}} \right) - 1} \right] \\ & \quad - I_{sd3} \times \left[ {\exp \left( {\frac{{q \times (V_{L} + R_{s} \times I_{L} )}}{{n_{3} \times k \times T}}} \right) - 1} \right] - \frac{{V_{L} + R_{s} \times I_{L} }}{{R_{sh} }} - I_{L} \\ \end{aligned}$$

### Ethical Statement

The manuscript has not been submitted to more than one journal for simultaneous consideration and has not been published elsewhere in any form or language.

## The proposed algorithm

This section shall elucidate the underlying principle of the RIME, along with the incorporated environment random interaction (ERI) strategy, the NMs mechanism, and the upgraded algorithm, referred to as ERINMRIME.

### RIME

#### Soft-rime search strategy

A highly stochastic process characterizes the growth of soft rime. Rime particles exhibit free movement across the surface of the attached objects, with a tendency to grow gradually in a specific direction. Therefore, a novel soft rime search strategy has been proposed to simulate the inherent randomness and extensive coverage of soft rime accurately. It enables RIME to perform global searches more comprehensively. The specific updating formula of rime is described as follows:

14 $$R_{ij}^{new} = R_{best,j} + r_{1} \cos \theta *\beta *(h*(Ub_{ij} - Lb_{i,j} ) + Lb_{i,j} ),r_{2} < E$$

where $R_{ij}^{new}$ is the new position of the rime particles updated. *I* represents the *i*th rime particle. *J* represents the *j*th dimension. $Ub_{i,j}$ and $Lb_{ij}$ represent the upper and lower bounds of the *i*th rime particle in the *j*th dimension, respectively. $R_{best,j}$ is the *j*th dimension of the optimal rime particles. $r_{1}$, $r_{2}$ and *h* are three random numbers in the range (-1, 1), independently. $\theta$, $\beta$ and *E* change with the number of iterations. Their specific definitions are as follows:

15 $$\theta = \pi *\frac{t}{10*T}$$

16 $$\beta = 1 - [\frac{w*t}{T}]/w$$

17 $$E = \sqrt {(t/T)}$$

where $t$ is the number of current iterations. *W* sets a constant, $w = 5$. *T* represents the maximum number of iterations.

#### Hard-rime puncture mechanism

In contrast to soft rime growth, the growth of hard rime is relatively straightforward and more regular. Each rime agent passes through easily due to the same direction of growth, which leads to the phenomenon known as rime puncture. RIME proposes the mechanism of hard rime puncture to accurately model the growth of hard rime when rime particles condense into this state. This mechanism enhances the local exploration ability of the RIME, by expanding the solution space and enabling more effective sampling of the local search area.

The specific updating formula of rime particles is shown below.

18 $$R_{ij}^{new} = R_{best,j} ,r_{3} < F^{normr} (S_{i} )$$

where $R_{ij}^{new}$ is the newly updated position of the rime particles. $r_{3}$ is a random number in the range (-1, 1). $R_{best,j}$ is the *j*th dimension of the optimal rime particles. $F^{normr} (S_{i} )$ is the normalized value of the current particle fitness value.

#### The positive greedy selection mechanism (PGSM)

**Figure 5:**
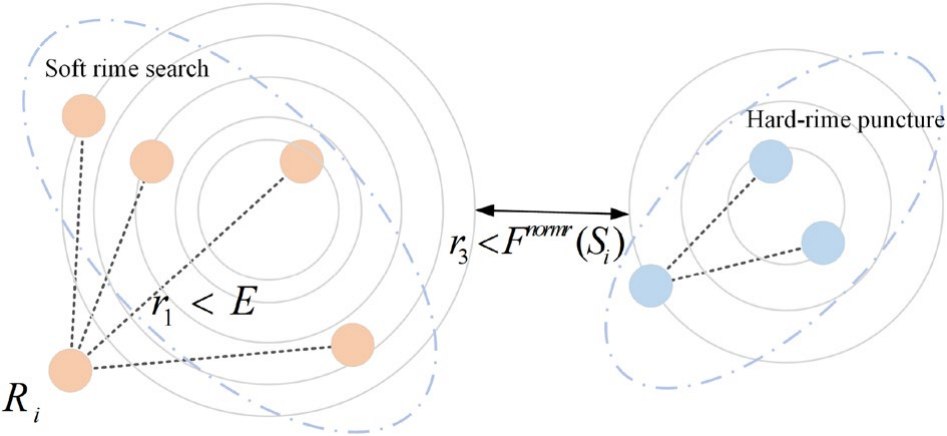
The mechanics of  RIME’s schematic diagram.

The PGSM is an improvement on the original greedy selection mechanism. The updated fitness value $F(R_{i}^{new} )$ of the particle is compared with that of the particle before the update $F(R_{i} )$. If the updated fitness value is better than that before the update, $F(R_{i} )$ will be replaced and the solution of two particles will be replaced. Eventually, the fitness value of the optimal solution $F(R_{best} )$ and the corresponding solution are updated. By incorporating the PGSM, the algorithm ensures that the population consistently progresses toward a globally optimal solution at every iteration. The schematic diagram of RIME is shown in Fig. 5.

19 $$\left\{ {\begin{array}{*{20}l} {F(R_{i} ) = F(R_{i}^{new} )} \hfill \\ {R_{i} = R_{i}^{new} } \hfill \\ \end{array} } \right.,F(R_{i}^{new} ) < F(R_{i} )$$

20 $$\left\{ {\begin{array}{*{20}l} {F(R_{best} ) = F(R_{i}^{new} ),} \hfill & {F(R_{i}^{new} ) < F(R_{i} )\& \& } \hfill \\ {R_{best} = R_{i}^{new} } \hfill & {F(R_{i}^{new} ) < F(R_{best} )} \hfill \\ \end{array} } \right.$$

### ERI strategy

**Figure 6:**
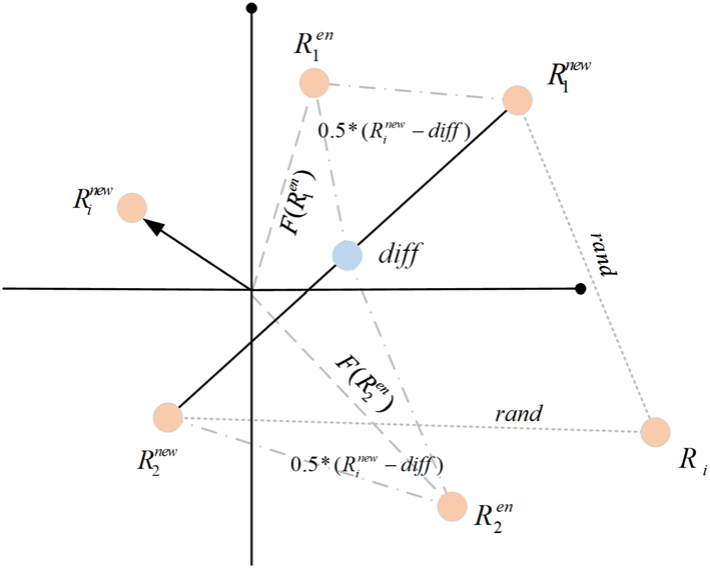
The mechanics of ERI’s schematic diagram.

This section introduced the ERI strategy, which is a global exploratory approach. Although RIME takes into account the different formation methods and morphologies of the two types of rime, the newly formed rime will change its form due to further changes in the environment. Rime particles are susceptible to changes in wind speed or temperature, which can cause them to undergo transformations and change into other forms of rime particles in the air. The RIME lacks further interaction with the environment after the formation of rime particles, which leads to the algorithm become stuck in local optima. To address this issue, this study introduces the ERI strategy^70^ to achieve further interaction with the environment. In this strategy, two random solutions are generated to control the exploration area of the algorithm. This strategy enables a thorough exploration of the surrounding regions of each individual within a certain distance, facilitating a global search effect throughout the entire algorithm. Therefore, the newly formed rime particles will undergo spontaneous modifications in response to environmental fluctuations. This ensures that the algorithm can quickly break free from local optima in a timely manner and explore the solution space more comprehensively. The following detailed description of the policy is a a full description of the policy. Fig. 6 displays the ERI strategy’s schematic diagram.

21 $$diff = \frac{{R_{1}^{new} + R_{2}^{new} }}{2}$$

22 $$R_{1}^{en} = 0.5*(R_{i}^{new} - diff)$$

23 $$R_{2}^{en} = 0.5*\left( {R_{i}^{new} + diff} \right)$$

where $R_{1}^{new}$ and $R_{2}^{new}$ are two particles randomly selected from the newly created rime particles. $diff$ represents the midpoint between the two random solutions. It controls the distance by which each individual further explores its surrounding area. The assignment of parameters $diff$ aids in guiding the exploration process, enabling the algorithm to effectively explore the solution space with a balanced approach. $R_{i}^{new}$ is the *i*th solution of the new solution. $R_{1}^{en}$ and $R_{2}^{en}$ represent the solution generated by two randomly selected particles after environmental interaction.

24 $$\left\{ {\begin{array}{*{20}l} {\begin{array}{*{20}l} {temp = F(R_{1}^{en} )} \hfill \\ {ss = R_{1}^{en} } \hfill \\ \end{array} ,} \hfill & {F(R_{1}^{en} ) < F(R_{2}^{en} )} \hfill \\ {\begin{array}{*{20}l} {temp = F(R_{2}^{en} )} \hfill \\ {ss = R_{2}^{en} } \hfill \\ \end{array} ,} \hfill & {F(R_{1}^{en} ) \ge F(R_{2}^{en} )} \hfill \\ \end{array} } \right.$$

25 $$R_{i}^{new} = ss,temp < F(R_{i}^{new} )$$

Comparing the fitness values of solutions generated through environmental interaction, the ERINMRIME selects the relatively optimal solution and eventually compares it with $F(R_{i}^{new} )$. If the *temp* is better, the algorithm updates $R_{i}^{new}$. This ensures that the solution is moving in the direction of the global optimal solution.

### NMs

**Figure 7:**
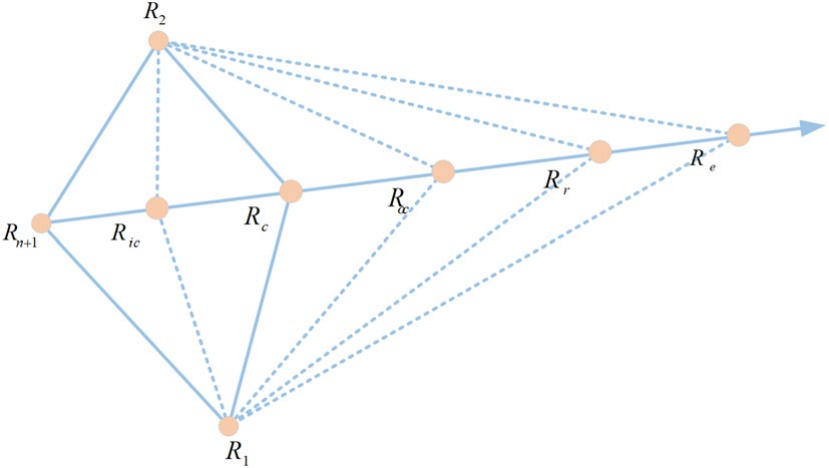
The mechanics of NMs’s schematic diagram.

The NMs mechanism^71^ is a well-established approach for solving optimization problems. Due to its remarkable local search capability, it has been extensively employed in the realm of PV model parameter identification. As a consequence, this study uses the NMs mechanism to boost the local exploitation capability of ERINMRIME. A simplex can be visualized as a polyhedron with $n + 1$ vertices, which forms the convex hull of n dimensions. The NMs mechanism utilizes this geometric concept to improve the current position of the solution by performing a sequence of operations such as reflection, expansion, and contraction, which ultimately leads to the formation of a new simplex with improved properties. By repeating this process iteratively, the NMs mechanism can efficiently search the solution space and converge toward the ideal solution. It continuously refines the solutions, guiding the algorithm to estimate the parameters in the PV models more accurately. The following are the actual steps of the NMs mechanism: Fig. 7 displays the schematic diagram of NMs.

1. *Choice*. Each individual is sorted according to fitness value and all individuals are numbered from 1 to *n* + 1. As shown in Eq. (26). The mechanism selects the optimal two individuals and averages them to get the position of the simplex centroid. The specific calculation formula is shown in Eq. (27).
  26 $$F(R_{1} ) \le F(R_{2} ) \le \cdots \le F(R_{n + 1} )$$
  27 $$R_{c} = \frac{{R_{1} + R_{2} }}{2}$$
2. *Reflection*. The reflection position $R_{r}$ is constructed by combining the center of mass $R_{c}$ and the worst solution $R_{n + 1}$. The specific formula is shown in Eq. (28).
  28 $$R_{r} = R_{c} + \alpha *(R_{c} - R_{n + 1} )$$
  where $\alpha$ is the reflection coefficient. If $F(R_{1} ) < F(R_{r} ) < F(R_{n} )$, the mechanism performs $R_{n + 1} = R_{r}$. In other words, if the newly generated reflection position is between the simplex optimal value and the subdifferential solution, it means that the worst solution has been promoted.
3. *Extension*. If $F(R_{r} ) > F(R_{1} )$, the exploration area is further expanded by combining the center of mass $R_{c}$ and the reflection position $R_{r}$. In other words, if $F(R_{r} ) > F(R_{1} )$, it indicates that the best way to find the global optimal solution is to search in the direction of reflection.
  29 $$R_{e} = R_{c} + \beta *(R_{r} - R_{c} )$$
  where $R_{e}$ is the expanded position. $\beta$ is the expansion coefficient. If $F(R_{e} ) < F(R_{r} )$, the mechanism performs $R_{n + 1} = R_{e}$. Otherwise, the mechanism performs $R_{n + 1} = R_{r}$. In other words, if the expanded position is the position of the centroid, it means that the expansion is valuable and the worst solution is updated. Otherwise, the newly generated position returns to the origin position. The worst solution is equal to the position of the centroid.
4. *Compression*. If $F(R_{n} ) < F(R_{r} ) < F(R_{n + 1} )$ is established, the mechanism executes Eq. (30).
  30 $$R_{oc} = R_{c} + \gamma *(R_{r} - R_{c} )$$
  where $\gamma$ is the shrinkage coefficient. If $F(R_{oc} ) < F(R_{r} )$, the mechanism makes $R_{n + 1} = R_{oc}$. Similar to expansion, if the contracted position is better than the centroid position, the worst solution is updated to the contracted position. Otherwise, the mechanism continues to execute step 5 to try internal shrinkage.
5. *Contraction*. If $F(R_{r} ) > F(R_{n + 1} )$ is true, the mechanism performs internal shrinkage according to **Eq. **(31).
  31 $$R_{ic} = R_{c} + \gamma *(R_{r} - R_{c} )$$

If $F(R_{oc} ) < F(R_{n + 1} )$ is established, the mechanism executes $R_{n + 1} = R_{oc}$.

### The proposed RIME-based algorithm

To address the existing limitations of the RIME, such as low convergence accuracy and the propensity to become stuck in local optima, this study has incorporated two novel strategies, namely the ERI strategy and the NMs mechanism. On the one hand, the ERI strategy leverages two random solutions to regulate the algorithm's ability to explore the world, safeguarding it against becoming stuck in local optima and enhancing its capacity for global search. On the other hand, the NMs mechanism employs a scaling transformation centered around the current best solution to focus on the local region and obtain higher-precision solutions, thereby bolstering the algorithm's capability for local exploitation. The amalgamation of these two mechanisms fosters a higher degree of equilibrium in the algorithm, substantially enhancing its convergence accuracy. ERINMRIME mainly comprises the following procedural steps:

- Step 1: Initialize the position of the rime particles using Eq. (32).
  32 $$R_{i} = rand \cdot (ub - lb) + lb,i = 1, \ldots ,N$$
  where *rand* is a random number in the range of (0, 1). *ub* and *lb* are the upper and lower boundaries respectively. *N* is the population size. $R_{i}$ represents the generated *i*th rime particle.
- Step 2: Fitness values are calculated for randomly generated rime populations to screen out the optimal particles.
- Step 3: At the commencement of each iteration, the algorithm executes the soft rime strategy, following the principles outlined in Eq. (14)–(17), which facilitates the updating of the rime population and helps the algorithm perform a global search.
- Step 4: The algorithm determines whether to employ the hard-rime puncture mechanism based on the conditions specified in Eq. (18). This mechanism aims to conduct further exploration within the local solution space, where a potential globally optimal solution may exist.
- Step 5: According to Eq. (19)–(20), the algorithm implements the PGSM to select the newly updated rime population to ensure that the population renewal is always moving in the right direction. The value of the optimal rime particle is updated at the same time.
- Step 6: According to Eq. (21)–(25), the ERI strategy of the environment is implemented. By means of the haphazard engagement with the surroundings, the rime particles in the solution space can be transformed randomly, which makes the algorithm explore the solution space more fully.
- Step 7: According to Eq. (26)—Eq. (31), the algorithm effectively executes the NMs mechanism. This mechanism guides the algorithm towards the global optimal solution, further enhancing solution quality through simplex transformations. Moreover, it promotes the local development capability of ERINMRIME, ultimately achieving a deeper level of balance in the algorithm's performance.
- Step 8: Iterate through the aforementioned process until the specified stopping condition is met.

**Figure 8:**
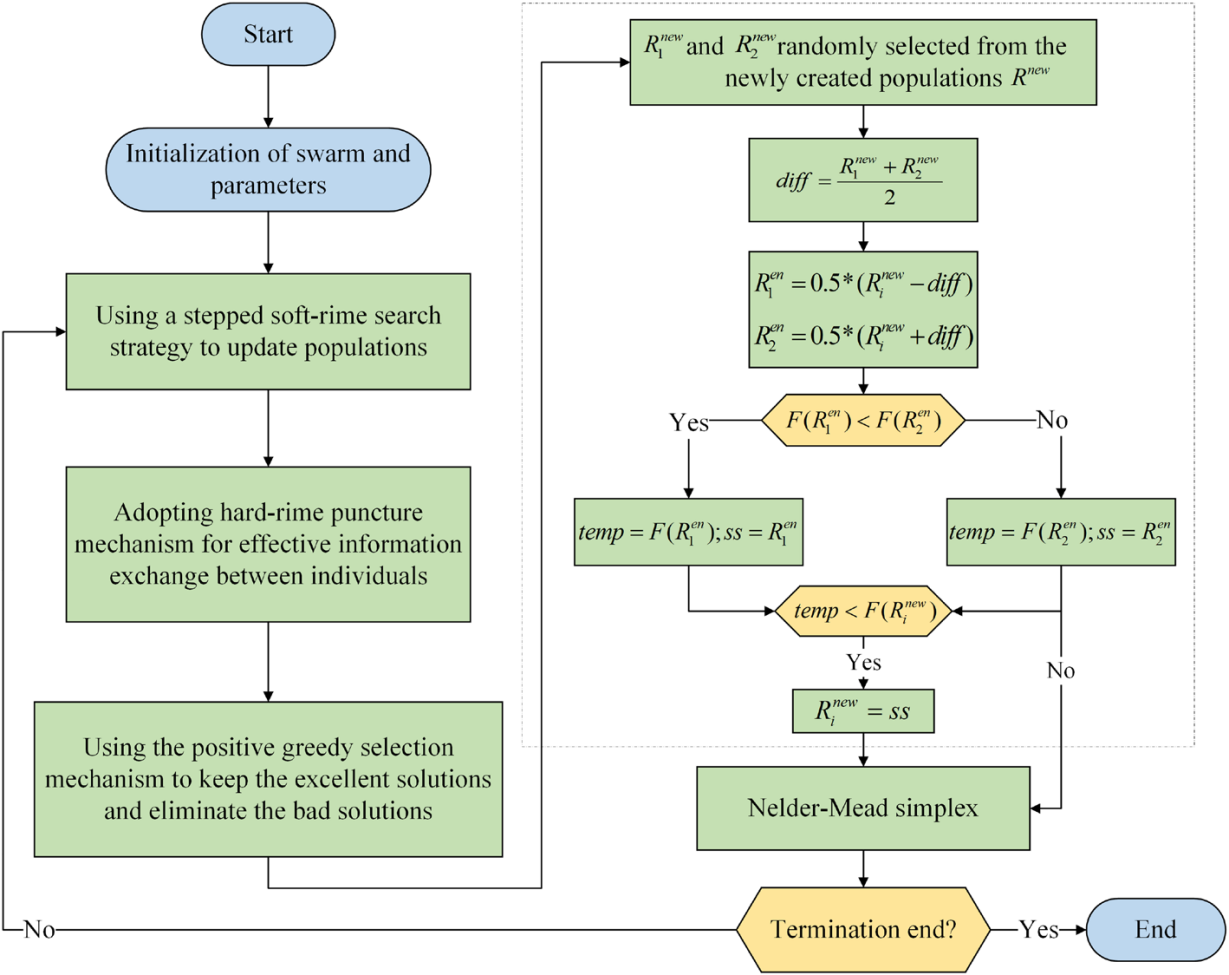
Flowchart of ERINMRIME.

To provide a clear illustration of the procedural flow of ERINMRIME, the algorithmic flowchart is depicted in Fig. 8, while the detailed pseudo-code of the algorithm is presented in Algorithm A1 (Online Appendix).

### Complexity analysis

Algorithm complexity analysis is a crucial step in evaluating and optimizing algorithm performance. It quantitatively assesses algorithm efficiency, resource requirements, and scalability, helping us make informed decisions in algorithm design and selection^72^.

In this study, the complexity of the proposed ERINMRIME was analyzed using the method proposed by Ni *et al*.^73^. The complexity of ERINMRIME is mainly determined by key factors such as the maximum number of iterations (*MaxIt*), population size (*N*), and problem dimension (*D*). The main components of ERINMRIME include the initialization of fitness values, RIME's original composition, NMs mechanism, and ERI strategy. The complexity of initializing fitness values is $$O(N)$$. The complexity of RIME is known to be $$O((N + \log N)*N*MaxIt)$$ based on the original paper. The complexity of the ERI strategy is $$O(N*MaxIt)$$. The complexity of the NMs mechanism is $$O(D*MaxIt)$$. Combining these complexities, the overall complexity of ERINMRIME is $$O(N + MaxIt*(N*(N + \log N) + N + D))$$.

## Experiments and analysis for benchmark functions

To validate the optimization performance of ERINMRIME, this study conducted experiments on the benchmark functions of IEEE CEC 2017 and IEEE CEC 2020. ERINMRIME was compared with other improved algorithms in these experiments.

### Experimental settings

#### IEEE CEC 2017 and IEEE CEC 2020

The experiments were carried out independently on IEEE CEC 2017 and IEEE CEC 2020 benchmark functions in order to guarantee impartiality and thoroughness.

**IEEE CEC 2017:** It consists of benchmark functions primarily categorized into three types. $F_{1} - F_{3}$ represents unimodal functions. $F_{4} - F_{10}$ represents multimodal functions. $F_{11} - F_{20}$ represents hybrid functions. $F_{21} - F_{30}$ represents composition functions (a combination of functions from different test problems)^74^. Table 1 comprehensively describes the 30 benchmark functions included in IEEE CEC 2017.

**Table 1:** An overview of the test functions for IEEE CEC 2017 (Search Range: [− 100, 100]^*D*^).

Class	No	Functions	Optimum
Unimodal Functions	1	Shifted and Rotated Bent Cigar Function	100
	2	Shifted and Rotated Bent Sum of Different Power Function*	200
	3	Shifted Rotated Zakharov Function	300
Multimodal Functions	4	Shifted and Rotated Rosenbrock’s Function	400
	5	Shifted and Rotated Rastrigin’s Function	500
	6	Shifted and Rotated Expanded Scaffer’s F6 Function	600
	7	Shifted and Rotated Lunacek Bi_Rastrigin’s Function	700
	8	Shifted and Rotated Non-Continuous Rastrigin’s Function	800
	9	Shifted and Rotated Levy Function	900
	10	Shifted and Rotated Schwefel’s Function	1000
Hybrid Functions	11	Hybrid Function 1 (*N* = 3)	1100
	12	Hybrid Function 2 (*N* = 3)	1200
	13	Hybrid Function 3 (*N* = 3)	1300
	14	Hybrid Function 4 (*N* = 4)	1400
	15	Hybrid Function 5 (*N* = 4)	1500
	16	Hybrid Function 6 (*N* = 4)	1600
	17	Hybrid Function 6 (*N* = 5)	1700
	18	Hybrid Function 6 (*N* = 5)	1800
	19	Hybrid Function 6 (*N* = 5)	1900
	20	Hybrid Function 6 (*N* = 6)	2000
Composition Functions	21	Composition Function 1 (*N* = 3)	2100
	22	Composition Function 2 (*N* = 3)	2200
	23	Composition Function 3 (*N* = 4)	2300
	24	Composition Function 4 (*N* = 4)	2400
	25	Composition Function 5 (*N* = 5)	2500
	26	Composition Function 6 (*N* = 5)	2600
	27	Composition Function 7 (*N* = 6)	2700
	28	Composition Function 8 (*N* = 6)	2800
	29	Composition Function 9 (*N* = 3)	2900
	30	Composition Function 10 (*N* = 3)	3000

All benchmark functions are formulated as unconstrained minimization problems. The mathematical model is represented as follows:

33 $$f(X),X = (x_{1} , \ldots ,x_{j} \ldots ,x_{D} ) \in S$$

where $f(X)$ is the objective function. *X* is the decision variable vector. $x_{j}$ represents the decision variable within the range $lb_{j} \le x_{j} \le ub_{j} ,j = (1, \ldots ,D)$. *D* represents the dimension. *S* is the decision space, which ranges from -100 to 100.

IEEE CEC 2020: This collection contains 10 benchmark functions. Table 2 provides a comprehensive description of the 10 benchmark functions included in IEEE CEC 2020.

**Table 2:** Overview of the test functions for IEEE CEC 2020 (Search Range: [− 100, 100]^*D*^).

Class	No	Functions	Optimum
Unimodal Functions	1	Shifted and Rotated Bent Cigar Function	100
Multimodal Functions	2	Shifted and Rotated Schwefel’s Function	1100
	3	Shifted and Rotated Lunacek bi-Rastrigin Function	700
	4	Expanded Rosenbrock’s plus Griewangk’s Function	1900
Hybrid Functions	5	Hybrid Function 1 (*N* = 3)	1700
	6	Hybrid Function 2 (*N* = 4)	1600
	7	Hybrid Function 3 (*N* = 5)	2100
Composition Functions	8	Composition Function 1 (*N* = 3)	2200
	9	Composition Function 2 (*N* = 4)	2400
	10	Composition Function 3 (*N* = 5)	2500

#### Measure metrics

To ensure a more reliable assessment of algorithm performance, this study employed various metrics such as average value (Avg) and standard deviation (Std). Convergence curves were also compared to analyze the experimental results. The best results among the tested experiments were highlighted in bold for easy identification. Moreover, non-parametric statistical tests were employed to ascertain the statistical significance of the enhanced algorithms, specifically the Wilcoxon Signed Rank Test (WSRT)^75^ and the Friedman Test (FT)^76^. The p-value was set to 0.05. A p-value of less than 0.05 suggests a significant difference between the two algorithms^77^. This significance level ensures that there is less than a five percent probability for test^78^.

### Parameter settings

Experiments were conducted using MATLAB R2018b software on a Windows 11 operating system to ensure fairness in the experiments. The hardware specifications included an Intel i7-12700H CPU and 16 GB of RAM. In addition, the effectiveness of the algorithm performance evaluation is based on the fairness and rationality of the experimental setting. Thus, the parameters for the experiments were kept consistent. The population size (*N*) was set to 30, the issue dimension (*D*) was 30, and the maximum iteration count (*MaxIt*) was set to 300,000. This implies that the search agent evaluations were performed a total of 300,000 times. The upper boundary (*ub*) and lower boundary (*lb*) of the search space were established at 100 and -100, correspondingly. Additionally, each function involved in the experiments was also independently tested 30 times.

The specific parameters of ERINMRIME proposed in this paper are directly adopted from the original RIME values. No new parameters were introduced in the ERI strategy and NMs mechanism.

### Experimental results on IEEE CEC 2017

To assess ERINMRIME’s optimization performance, this section compared it to other improved algorithms using the IEEE CEC 2017 benchmark functions of IEEE CEC 2017. The comparison algorithms including m_SCA, SCADE, ASCA_PSO, OBSCA, HGWO, RCBA, and CBA. The parameter settings for each algorithm are provided in Table 3.

**Table 3:** Parameter settings for comparison algorithms.

Methods	Other Parameters
SCADE^79^	*beta_min* = 0.2; *beta_max* = 0.8; *pCR* = 0.8
ASCA_PSO^80^	*M* = 4; *N* = 9; *Vmax* = 6; *wMax* = 0.9;
	*wMin* = 0.2; $c_{1} = 2$;$c_{2} = 2$
HGWO^81^	$\beta \min = 0.2;$ $\beta \max = 0.8;$ *pCR* = 0.2
CAGWO^82^	*type* = 2; *a* = [0 2]
m_SCA^83^	$JR = 0.1$; $a = 2$
RCBA^84^	*Qmin* = 0; *Qmax* = 2
CBA^85^	*Qmin* = 0; *Qmax* = 2

Table 4 presents the average values and standard deviations of 8 algorithms. ERINMRIME performs poorly on composite functions in the table but achieves the best or near-best average values on the remaining benchmark functions. Considering the comparison results of ERINMRIME with the other seven algorithms in terms of WSRT and overall ranking in Table 5, ERINMRIME ranks first overall, indicating its significant optimization capability. Generally speaking, WSRT whether the algorithms have statistical significance in their test functions. In Table 5, symbols "+/=/−" indicate whether ERINMRIME is superior to, equal to, or lower than other algorithms, respectively. ERINMRIME obtains a WSRT value of 3.43 and significantly outperforms other improved algorithms on over half of the benchmark functions out of the 30. This indicates that ERINMRIME significantly differs from the other seven algorithms on most functions.

**Table 4:** Avg and Std of ERINMRIME compared with 7 algorithms on IEEE CEC 2017. Significant values are in bold.

	F1		F2		F3	
	Avg	Std	Avg	Std	Avg	Std
ERINMRIME	**2.6880e+02**	**6.4216e + 02**	3.5389e+05	1.2745e+06	**3.0000e+02**	**7.8009e-04**
m_SCA	1.1222e+10	4.8361e+09	1.4320e+33	4.0281e+33	4.1747e+04	6.9354e+03
SCADE	2.7627e+10	3.8456e+09	2.5253e+35	4.2575e+35	6.1279e+04	5.7888e+03
ASCA_PSO	2.4812e+09	2.7734e+09	5.2918e+27	2.8970e+28	1.2561e+03	4.5310e+02
OBSCA	2.7062e+10	5.2229e+09	1.1180e+35	2.7989e+35	5.4617e+04	7.1061e+03
HGWO	1.0578e+10	1.1374e+09	4.3750e+30	1.5524e+31	7.1761e+04	5.6545e+03
RCBA	1.7595e+04	5.9320e+03	**2.2370e+02**	**3.4034e+01**	3.0056e+02	2.0531e-01
CBA	1.0140e+05	5.3453e+05	6.5878e+03	1.2502e+04	3.1402e+02	7.2449e+00

	F4		F5		F6	
	Avg	Std	Avg	Std	Avg	Std
ERINMRIME	**4.0251e+02**	**1.1564e+01**	7.7377e+02	5.5673e+01	6.5099e+02	9.3614e+00
m_SCA	9.5003e+02	2.0323e+02	**6.6595e+02**	2.6554e+01	6.3203e+02	7.1717e+00
SCADE	2.4101e+03	6.1615e+02	7.9486e+02	**1.1666e+01**	6.5795e+02	5.5730e+00
ASCA_PSO	5.8387e+02	1.3486e+02	6.9159e+02	3.1809e+01	6.3160e+02	1.0109e+01
OBSCA	2.2642e+03	5.1228e+02	7.7463e+02	1.8110e+01	6.4628e+02	5.6457e+00
HGWO	9.3288e+02	6.4607e+01	7.0578e+02	1.4637e+01	**6.3096e+02**	**2.3525e+00**
RCBA	4.7094e+02	4.1396e+01	7.5275e+02	5.7695e+01	6.6712e+02	1.0886e+01
CBA	5.1078e+02	3.3261e+01	7.4458e+02	6.2852e+01	6.6848e+02	1.1584e+01

	F7		F8		F9	
	Avg	Std	Avg	Std	Avg	Std
ERINMRIME	9.9932e+02	9.5876e+01	1.1006e+03	5.1436e+01	1.0563e+04	2.0408e+03
m_SCA	1.1358e+03	7.4733e+01	**9.8126e+02**	2.8227e+01	5.4598e+03	1.4329e+03
SCADE	1.2814e+03	5.2143e+01	1.1190e+03	2.0152e+01	1.0172e+04	9.6680e+02
ASCA_PSO	**9.9925e+02**	3.3962e+01	1.0157e+03	3.1870e+01	5.3085e+03	1.9031e+03
OBSCA	1.2861e+03	5.2863e+01	1.0968e+03	2.4843e+01	8.5349e+03	1.0871e+03
HGWO	1.0620e+03	**3.3649e+01**	1.0153e+03	**1.2997e+01**	**4.5716e+03**	**4.5840e+02**
RCBA	2.0111e+03	2.9956e+02	1.1270e+03	7.1250e+01	9.6164e+03	3.5836e+03
CBA	1.9657e+03	3.2173e+02	1.1298e+03	6.0277e+01	8.5041e+03	2.4936e+03

	F10		F11		F12	
	Avg	Std	Avg	Std	Avg	Std
ERINMRIME	**5.1314e+03**	7.7080e+02	**1.3690e+03**	1.0587e+02	**6.0464e+03**	**7.2156e+03**
m_SCA	5.6797e+03	7.0925e+02	3.1586e+03	1.4682e+03	2.2001e+08	1.4217e+08
SCADE	7.3372e+03	**3.7706e+02**	6.7487e+03	1.1248e+03	2.6847e+09	7.2949e+08
ASCA_PSO	5.7796e+03	7.8072e+02	1.3942e+03	**5.3619e+01**	1.8745e+08	1.8293e+08
OBSCA	6.9951e+03	4.0839e+02	5.4094e+03	8.1185e+02	2.6508e+09	1.0525e+09
HGWO	6.6563e+03	3.9719e+02	6.4607e+03	1.3111e+03	8.3112e+08	2.1836e+08
RCBA	5.5161e+03	6.3547e+02	1.3856e+03	9.8347e+01	3.7267e+06	2.1323e+06
CBA	5.8283e+03	6.9337e+02	1.5093e+03	1.8355e+02	4.5278e+07	1.6844e+07

	F13		F14		F15	
	Avg	Std	Avg	Std	Avg	Std
ERINMRIME	**3.5486e+03**	**2.3720e+03**	**1.7987e+03**	**1.3213e+02**	**1.6514e+03**	**6.4079e+01**
m_SCA	4.9953e+07	8.1236e+07	2.6020e+05	1.9584e+05	3.2336e+06	1.2348e+07
SCADE	1.9240e+08	4.6105e+07	4.5163e+05	2.3224e+05	6.6361e+06	3.4335e+06
ASCA_PSO	5.4315e+06	1.5750e+06	4.2527e+04	3.0515e+04	1.0186e+06	4.2335e+05
OBSCA	2.3619e+08	1.4235e+08	3.7676e+05	2.5081e+05	1.4986e+07	1.7575e+07
HGWO	9.6247e+07	3.8528e+07	5.8968e+05	3.6992e+05	5.5830e+06	2.8017e+06
RCBA	7.1049e+04	5.2902e+04	3.8246e+03	2.9584e+03	3.1175e+04	2.3810e+04
CBA	9.4257e+04	8.3535e+04	4.2504e+04	3.3221e+04	8.8912e+04	9.7751e+04

	F16		F17		F18	
	Avg	Std	Avg	Std	Avg	Std
ERINMRIME	3.0006e+03	3.5431e+02	2.5164e+03	2.5268e+02	**2.0842e+03**	**2.0917e+02**
m_SCA	**2.6268e+03**	2.9215e+02	**2.2423e+03**	2.3983e+02	8.7476e+05	8.3771e+05
SCADE	3.5666e+03	1.9452e+02	2.6724e+03	1.8790e+02	2.4797e+06	1.2657e+06
ASCA_PSO	2.7568e+03	2.8411e+02	2.3371e+03	2.2093e+02	2.9207e+05	1.7903e+05
OBSCA	3.5497e+03	2.9595e+02	2.6181e+03	1.9962e+02	2.8897e+06	1.4796e+06
HGWO	2.9831e+03	**1.8297e+02**	2.2818e+03	**1.1286e+02**	1.2192e+06	9.1399e+05
RCBA	3.0207e+03	3.5819e+02	2.8652e+03	2.5501e+02	6.6369e+04	3.6469e+04
CBA	3.5104e+03	6.1652e+02	2.8813e+03	3.4692e+02	9.9168e+04	9.4196e+04

	F19		F20		F21	
	Avg	Std	Avg	Std	Avg	Std
ERINMRIME	9.6138e+03	**6.3397e+03**	2.6669e+03	2.2528e+02	**2.1173e+03**	3.5264e+01
m_SCA	8.4645e+05	1.8058e+06	2.5292e+03	1.7384e+02	2.7713e+03	5.2949e+02
SCADE	1.8100e+07	1.4547e+07	2.8713e+03	8.9826e+01	4.6675e+03	6.7328e+02
ASCA_PSO	1.9169e+06	2.0717e+06	**2.5003e+03**	1.3218e+02	2.2675e+03	1.1099e+02
OBSCA	1.8335e+07	1.5877e+07	2.7564e+03	**8.9474e+01**	4.2744e+03	8.4417e+02
HGWO	6.8690e+06	2.2749e+06	2.6180e+03	1.3402e+02	2.6631e+03	5.5741e+01
RCBA	**9.6075e+03**	7.3942e+03	2.9107e+03	2.4496e+02	2.1890e+03	**2.7372e+01**
CBA	4.4275e+05	3.2578e+05	2.9891e+03	2.0547e+02	2.2299e+03	3.4168e+01

	F22		F23		F24	
	Avg	Std	Avg	Std	Avg	Std
ERINMRIME	2.4917e+03	4.9430e+01	4.0720e+03	4.2925e+02	3.3736e+03	3.8969e+02
m_SCA	**2.3712e+03**	2.9655e+01	**3.0498e+03**	7.6182e+01	3.4672e+03	1.6952e+02
SCADE	2.5177e+03	1.6997e+01	3.1207e+03	3.3867e+02	**2.6000e+03**	**0.0000e+00**
ASCA_PSO	2.4002e+03	3.5405e+01	3.0718e+03	5.5289e+01	3.5011e+03	2.7633e+02
OBSCA	2.4930e+03	2.1846e+01	3.3516e+03	6.2967e+01	2.6010e+03	4.4532e+00
HGWO	2.4278e+03	**1.1565e+01**	3.0835e+03	**4.4003e+01**	3.1589e+03	4.3712e+02
RCBA	2.4748e+03	4.7322e+01	3.6293e+03	3.3415e+02	2.9078e+03	5.6989e+02
CBA	2.4678e+03	7.1956e+01	3.4787e+03	1.9095e+02	3.0955e+03	7.1146e+02

	F25		F26		F27	
	Avg	Std	Avg	Std	Avg	Std
ERINMRIME	2.9381e+03	3.4119e+01	7.1022e+03	1.7853e+03	4.8921e+03	4.5963e+02
m_SCA	3.3877e+03	2.7730e+02	6.5163e+03	5.2653e+02	3.8879e+03	1.5058e+02
SCADE	**2.7000e+03**	**0.0000e+00**	**2.8000e+03**	**0.0000e+00**	3.8892e+03	3.5304e+02
ASCA_PSO	2.9953e+03	6.6347e+01	6.8830e+03	1.3267e+03	3.8558e+03	1.1112e+02
OBSCA	2.7713e+03	1.7791e+02	2.8028e+03	7.7556e+00	4.2066e+03	**8.5662e+01**
HGWO	3.3885e+03	5.0543e+01	3.5834e+03	1.3985e+03	**3.6357e+03**	5.3705e+02
RCBA	3.0104e+03	1.0353e+02	5.0946e+03	3.5096e+03	4.0197e+03	2.1232e+02
CBA	3.0280e+03	4.7727e+01	5.2260e+03	3.5885e+03	3.8832e+03	1.7160e+02

	F28		F29		F30	
	Avg	Std	Avg	Std	Avg	Std
ERINMRIME	3.3093e+03	3.6247e+02	4.2232e+03	2.8738e+02	1.1358e+04	5.1025e+03
m_SCA	5.3602e+03	3.0992e+02	3.9669e+03	2.2378e+02	4.2927e+06	1.1247e+07
SCADE	**3.0000e+03**	**0.0000e+00**	**3.1000e+03**	**0.0000e+00**	2.3854e+05	3.9630e+05
ASCA_PSO	3.7966e+03	7.7165e+02	4.1369e+03	2.7222e+02	5.7336e+06	3.5259e+06
OBSCA	3.8620e+03	6.4901e+02	3.6316e+03	1.4799e+02	5.0612e+06	1.3013e+07
HGWO	3.9016e+03	2.0651e+02	3.1305e+03	6.3056e+01	**3.2000e+03**	**0.0000e+00**
RCBA	3.3590e+03	5.2841e+02	4.7273e+03	4.5161e+02	3.0016e+05	2.0347e+05
CBA	3.5005e+03	6.8302e+02	4.7860e+03	4.3488e+02	2.5197e+06	1.4710e+06

**Table 5:** WSRT of ERINMRIME compared with 7 algorithms on IEEE CEC 2017. Significant values are in bold.

	Overall Rank		
	RANK	+/=/−	AVG
**ERINMRIME**	**1**	**~**	**3.43**
m_SCA	4	17/1/12	4.2
SCADE	7	19/4/7	5.83
ASCA_PSO	2	15/5/10	3.7
OBSCA	8	17/5/8	5.87
HGWO	5	16/2/12	4.33
RCBA	3	17/8/5	4.0
CBA	6	22/4/4	4.63

**Figure 9:**
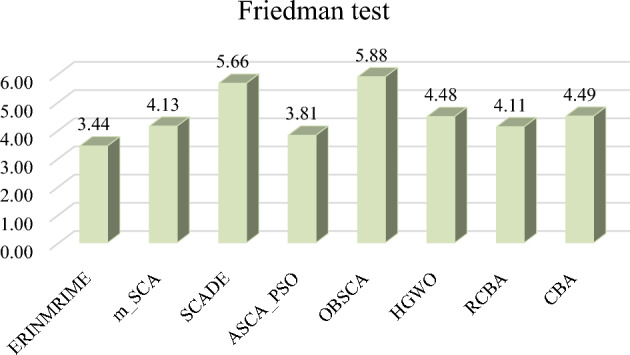
The outcome of ERINMRIME’s FT in comparison to seven algorithms at IEEE CEC 2017.

**Figure 10:**
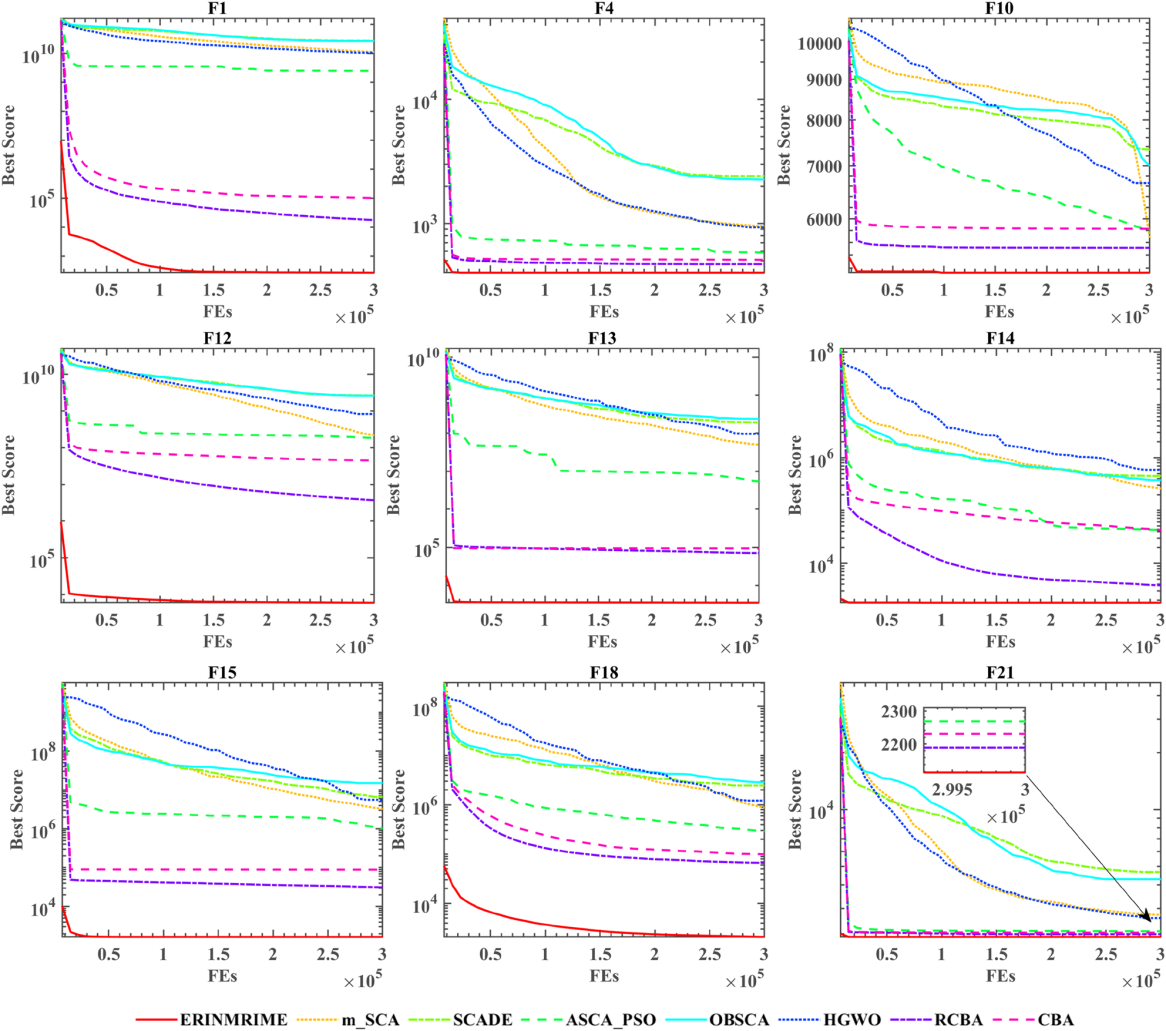
ERINMRIME’s convergence curves in comparison to seven different algorithms at IEEE CEC 2017.

Figure 9 depicts the bar chart of the FT rankings of the 8 algorithms. The p-value of ERINMRIME is 3.44, which is the lowest compared to other improved algorithms. FT can assess the stability of the algorithm's optimization performance. Therefore, this also indicates that ERINMRIME exhibits a certain level of stability. Figure 10 shows the convergence curves of ERINMRIME and the other 7 algorithms. From the red line in the graph, ERINMRIME exhibits the maximum convergence accuracy, as evidenced by its persistent placement at the highest convergence accuracy. Furthermore, the steepness of the red curve’s descent in the graph illustrates that ERINMRIME has the fastest convergence speed, allowing it to converge toward the optimal solution rapidly. To sum up, ERINMRIME can be proven to be an effective optimization algorithm, possessing stable optimization performance and significant competitiveness.

### Experimental results on IEEE CEC 2020

To further verify the algorithm’s optimization efficiency in this section, ERINMRIME is compared with other improved algorithms on the IEEE CEC 2020 benchmark functions of IEEE CEC 2020. The average values and standard deviations for each of the eight algorithms are shown in Table 6. Within the table, ERINMRIME achieves the optimal average value on half of the 10 benchmark functions in IEEE CEC 2020. This demonstrates the excellent optimization capability of this algorithm. Table 7 presents the WSRT values and overall rankings of ERINMRIME compared to the other 7 algorithms. ERINMRIME has a WSRT value of 2.3, which is the lowest among them, indicating a significant superiority of ERINMRIME compared to other algorithms.

**Table 6:** Avg and Std of ERINMRIME compared with 7 algorithms on IEEE CEC 2020. Significant values are in bold.

	F1		F2		F3	
	Avg	Std	Avg	Std	Avg	Std
ERINMRIME	**3.5484e+02**	**7.7744e+02**	**5.3177e+03**	7.1035e+02	9.9183e+02	9.2490e+01
m_SCA	1.0793e+10	5.3098e+09	5.8740e+03	8.2021e+02	1.1208e+03	7.0851e+01
SCADE	2.8299e+10	3.3876e+09	8.2652e+03	2.5859e+02	1.2768e+03	4.2216e+01
ASCA_PSO	1.8049e+09	2.0840e+09	5.9109e+03	7.6388e+02	**9.9004e+02**	**3.0407e+01**
OBSCA	2.7202e+10	5.9376e+09	7.3415e+03	**3.4766e+02**	1.2843e+03	5.7789e+01
HGWO	1.0828e+10	1.0866e+09	6.4214e+03	4.3865e+02	1.0324e+03	3.7902e+01
RCBA	1.7000e+04	6.4704e+03	5.7391e+03	6.5356e+02	1.9433e+03	2.8844e+02
CBA	3.2324e+03	3.6897e+03	5.7901e+03	6.8411e+02	2.0610e+03	2.8690e+02

	F4		F5		F6	
	Avg	Std	Avg	Std	Avg	Std
ERINMRIME	**7.6349e+03**	**5.3919e+03**	**1.5087e+04**	**1.8946e+04**	2.9414e+03	3.3409e+02
m_SCA	4.2492e+06	1.0096e+07	4.9670e+06	4.2226e+06	2.8045e+03	3.0504e+02
SCADE	1.7512e+07	1.5480e+07	1.4476e+07	5.3576e+06	3.5299e+03	2.1715e+02
ASCA_PSO	1.3565e+06	7.8002e+05	9.5460e+05	7.5662e+05	**2.8028e+03**	2.3836e+02
OBSCA	2.2451e+07	1.4708e+07	1.2446e+07	6.7792e+06	3.5248e+03	1.8417e+02
HGWO	7.0930e+06	3.8222e+06	6.4854e+06	3.4939e+06	3.0533e+03	**1.7348e+02**
RCBA	1.1446e+04	9.8265e+03	1.1968e+05	6.4565e+04	3.1927e+03	3.9729e+02
CBA	3.0594e+05	2.6072e+05	2.5948e+05	1.7983e+05	3.3884e+03	4.2481e+02

	F7		F8		F9	
	Avg	Std	Avg	Std	Avg	Std
ERINMRIME	**2.5706e+04**	**2.5470e+04**	2.4806e+03	5.9259e+01	3.3727e+03	3.9298e+02
m_SCA	8.9544e+05	8.1180e+05	**2.3748e+03**	1.9600e+01	3.5496e+03	1.2178e+02
SCADE	2.3297e+06	1.0364e+06	2.5126e+03	1.9280e+01	**2.6000e+03**	**0.0000e+00**
ASCA_PSO	2.8777e+05	2.2762e+05	2.4186e+03	3.6434e+01	3.3983e+03	4.2029e+02
OBSCA	2.1717e+06	1.3632e+06	2.4890e+03	2.7244e+01	2.6065e+03	1.4551e+01
HGWO	1.9169e+06	1.3283e+06	2.4257e+03	**1.3382e+01**	3.2523e+03	4.0916e+02
RCBA	7.1845e+04	4.6768e+04	2.4950e+03	6.8667e+01	2.9355e+03	5.6955e+02
CBA	1.0794e+05	5.0762e+04	2.5098e+03	7.4252e+01	2.9903e+03	6.1091e+02

	F10					
	Avg			Std		
ERINMRIME	2.9523e+03			6.4277e+01		
m_SCA	3.5022e+03			1.8170e+02		
SCADE	**2.7000e+03**			**0.0000e+00**		
ASCA_PSO	2.9830e+03			4.6835e+01		
OBSCA	2.7312e+03			7.5060e+01		
HGWO	3.3242e+03			2.1688e+02		
RCBA	3.0296e+03			5.0482e+01		
CBA	3.0228e+03			7.1993e+01

**Table 7:** WSRT of ERINMRIME compared with 7 algorithms on IEEE CEC 2020.  Significant values are in bold.

	Overall rank		
	RANK	+/=/−	AVG
**ERINMRIME**	**1**	** ~ **	**2.3**
m_SCA	5	7/2/1	4.7
SCADE	8	8/0/2	6.2
ASCA_PSO	2	6/3/1	3.6
OBSCA	7	7/1/2	5.8
HGWO	6	7/2/1	5.2
RCBA	3	6/3/1	3.8
CBA	4	8/1/1	4.4

**Figure 11:**
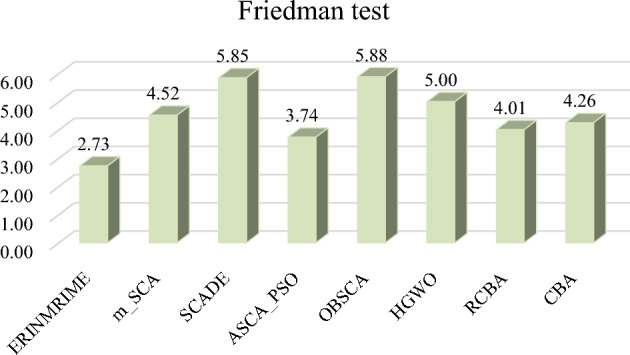
The outcome of the FT of ERINMRIME on IEEE CEC 2020 in comparison with seven methods.

**Figure 12:**
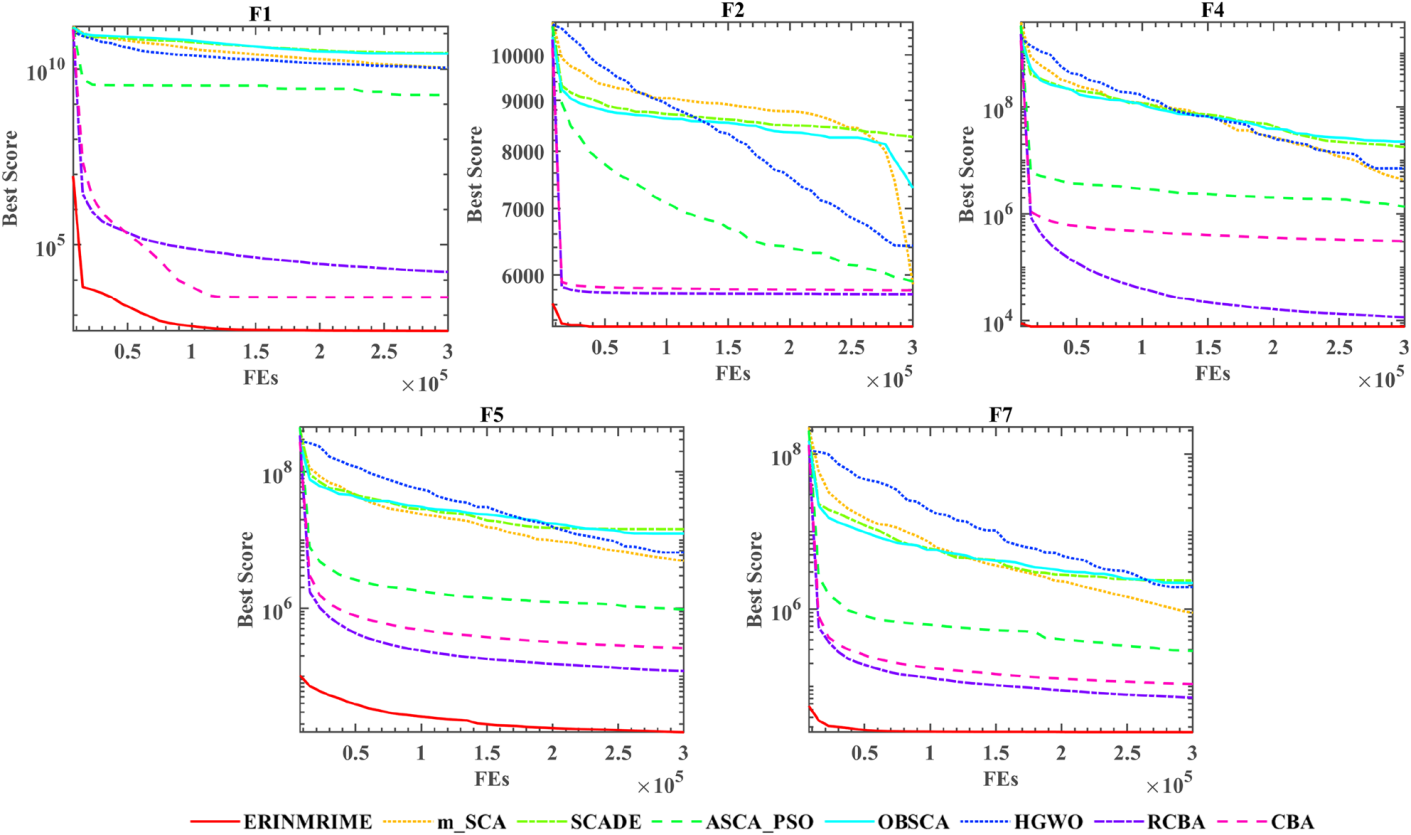
Convergence curves of ERINMRIME compared with 7 algorithms on IEEE CEC 2020.

Figure 11 represents the bar chart of the FT rankings of the 8 algorithms. ERINMRIME possesses the lowest FT index among them in the graph, indicating the stable optimization performance of this algorithm. Figure 12 displays the convergence curves of ERINMRIME with the other 7 algorithms. The red curve in these convergence plots shows that ERINMRIME consistently remains at the bottom of the graph, exhibiting the highest convergence accuracy and a fast convergence speed. In conclusion, ERINMRIME demonstrates unique competitiveness and significant optimization performance, whether in IEEE CEC 2017 or 2020.

## Experimental and analysis for PV models

To assess the effectiveness and reliability of ERINMRIME for parameter identification of PV models, a series of experiments are conducted on ERINMRIME, comparing it with other enhanced algorithms in the context of SDM, DDM, TDM, and the PV module model. Subsequently, to provide a more robust demonstration of ERINMRIME's efficacy in practical scenarios, this study conducts further testing of the algorithm using three commercial PV models.

In this study, the experiments were all implemented on MATLAB R2018b software. The operating system was Windows 10. The processor was Intel i7-6700HQ (3.40 GHz), and the memory was 16 GB. The basic parameters of the experiment were the same, the population size (*N*) was 30, the dimension (*dim*) of the problem was 30, the maximum number of iterations (*MaxIt*) was set to 20,000, and each involved function was independently tested for 30 times. This fair experimental setting reduces the impact of test environment deviation on the final experimental results. In the experimental setup, Table 8 presents the defined upper and lower bounds for each parameter. The *RMSE* is adopted as the evaluation metric to quantify the disparity between predicted and actual values. Moreover, the symbol " + " indicates that ERINMRIME outperforms the compared algorithms, while "=" denotes equivalent performance. Throughout the experimental process, the Absolute Error (*IAE*) and Relative Error (*RE*) are calculated in order to evalute and easure the differences between the experimental data and the estimated data. The expressions for $I_{IAE}$ and $I_{RE}$, $P_{IAE}$ and $P_{RE}$ can be written as follows:

34 $$I_{IAE} = \left| {I_{{{\text{measure}}}} - I_{simulate} } \right|$$

35 $$I_{RE} = \frac{{I_{measure} - I_{simulate} }}{{I_{measure} }} \times 100\%$$

36 $$P_{IAE} = \left| {P_{{{\text{measure}}}} - P_{simulate} } \right|$$

37 $$P_{RE} = \frac{{P_{measure} - P_{simulate} }}{{P_{measure} }} \times 100\%$$

where $I_{measure}$ represents the actual current value. $I_{simulate}$ represents the estimated current value. $P_{measure}$ indicates the actual power value. $P_{simulate}$ and indicates the estimated power value.

**Table 8:** The boundary of the unknown parameters.

Parameters	SDM/DDM/TDM		PV module model	
	Lower bound	Upper bound	Lower bound	Upper bound
$I_{ph} (A)$	0	1	0	2
$I_{sd} (\mu A)$	0	1	0	50
$R_{s} (\Omega )$	0	0.5	0	2
$R_{sh} (\Omega )$	0	100	0	2000
$n$	1	2	1	50
$I_{sd1} (\mu A)$	0	1	0	50
$I_{sd2} (\mu A)$	0	1	0	50
$n_{1}$	1	2	1	50
$n_{2}$	1	2	1	50

### Results on the SDM case

**Figure 13:**
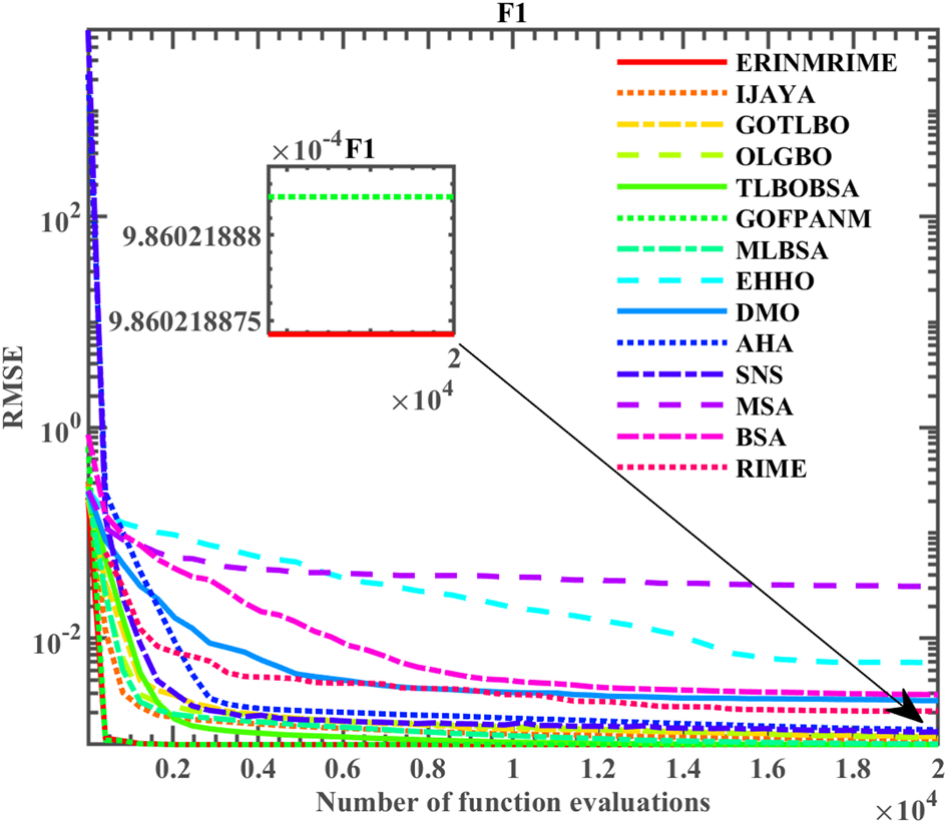
The convergence curve on the SDM.

This study compares ERINMRIME with other advanced algorithms, including IJAYA^86^, GOTLBO^87^, OLGBO^88^, TLBOBSA^64^, GOFPANM^89^, MLBSA^90^, EHHO^91^, Dwarf Mongoose Optimizer (DMO)^92^, Artificial Hummingbird Algorithm (AHA)^93^, Social Network Search (SNS)^94^, Mantis Search Algorithm (MSA)^95^, BSA^96^, RIME^44^. Particular experimental outcomes are displayed in Table 9. The *RMSE* by ERINMRIME significantly decreases compared to the RIME. The phenomenon indicates that the accuracy of ERINMRIME has been markedly promoted. Furthermore, ERINMRIME achieved an *RMSE* value of 9.86022E-04, comparable to the most recent advancements in algorithmic improvements, including OLGBO, TLBOBSA, GOFPANM, and MLBSA. This also reflects that ERINMRIME is worthy of deep exploration in parameter extraction of SDM, which has excellent development potential. To observe the benefits and drawbacks of the comparison method more intuitively, the image of ERINMRIME with its comparison algorithm on the *RMSE* standard is shown in Fig. 13. It is apparent that in the algorithm’s first phase, the convergence speed of ERINMRIME is second only to GOFPANM. But when more assessments are made, ERINMRIME progressively approaches and eventually surpasses GOFPANM, yielding the optimal result. Meanwhile, the image clearly illustrates that the purple curve of RIME quickly falls into a local optimum, indicating limited optimization capability. However, the red curve representing ERINMRIME successfully avoids local optima, demonstrating a substantial enhancement in the algorithm's optimization capabilities compared to the original RIME. In conclusion, ERINMRIME demonstrates outstanding performance and absolute superiority in the extraction of SDM parameters.

**Table 9:** Parameter extraction results of ERINMRIME on SDM.  Significant values are in bold.

Item	$I_{ph} (A)$	$I_{sd} (\mu A)$	$R_{s} (\Omega )$	$R_{sh} (\Omega )$	*n*	*RMSE*	Compare
ERINMRIME	0.760776	3.23E−07	0.036377	53.71764	1.48118	**9.86022E−04**	
IJAYA	0.760831	3.32E−07	0.036268	53.80931	1.484028	9.88467E−04	+
GOTLBO	0.760703	3.3E−07	0.036314	55.96758	1.483385	9.92120E−04	+
OLGBO	0.760775	3.23E−07	0.036377	53.71889	1.481183	**9.86022E−04**	=
TLBOBSA	0.760776	3.23E−07	0.036377	53.71852	1.481184	**9.86022E−04**	=
GOFPANM	0.760776	3.23E−07	0.036377	53.71852	1.481184	**9.86022E−04**	=
MLBSA	0.760776	3.23E−07	0.036377	53.71851	1.481184	**9.86022E−04**	=
EHHO	0.760772	3.69E−07	0.035801	57.42219	1.494743	1.02351E−03	+
DMO	0.760558	3.68E−07	0.035842	58.91412	1.481503	1.02577E−03	+
AHA	0.760775	3.24E−07	0.036366	53.76364	1.481412	1.36282E−03	+
SNS	0.760777	3.23E−07	0.036383	53.65636	1.481023	1.30237E−03	+
MSA	0.763318	7.97E−08	0.043429	81.4027	1.351823	3.06575E−02	+
BSA	0.761070	3.98E−07	0.035209	52.46628	1.502676	1.18624E−03	+
RIME	0.760313	3.33E−07	0.036326	60.06902	1.484159	1.02962E−03	+

**Figure 14:**
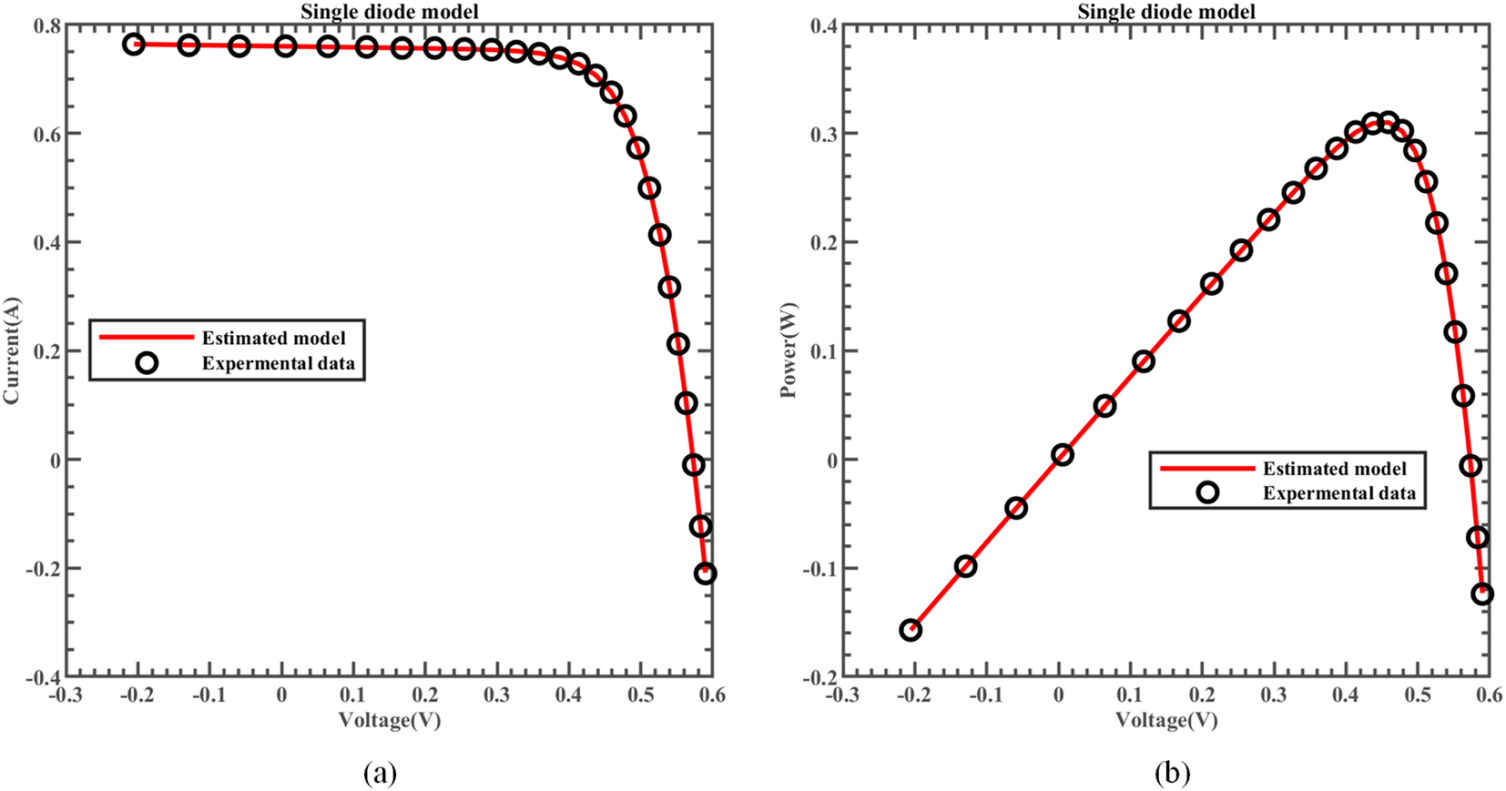
(**a**) The I-V curve of SDM simulated by ERINMRIME (**b**) The P–V graph gained by ERINMRIME.

**Figure 15:**
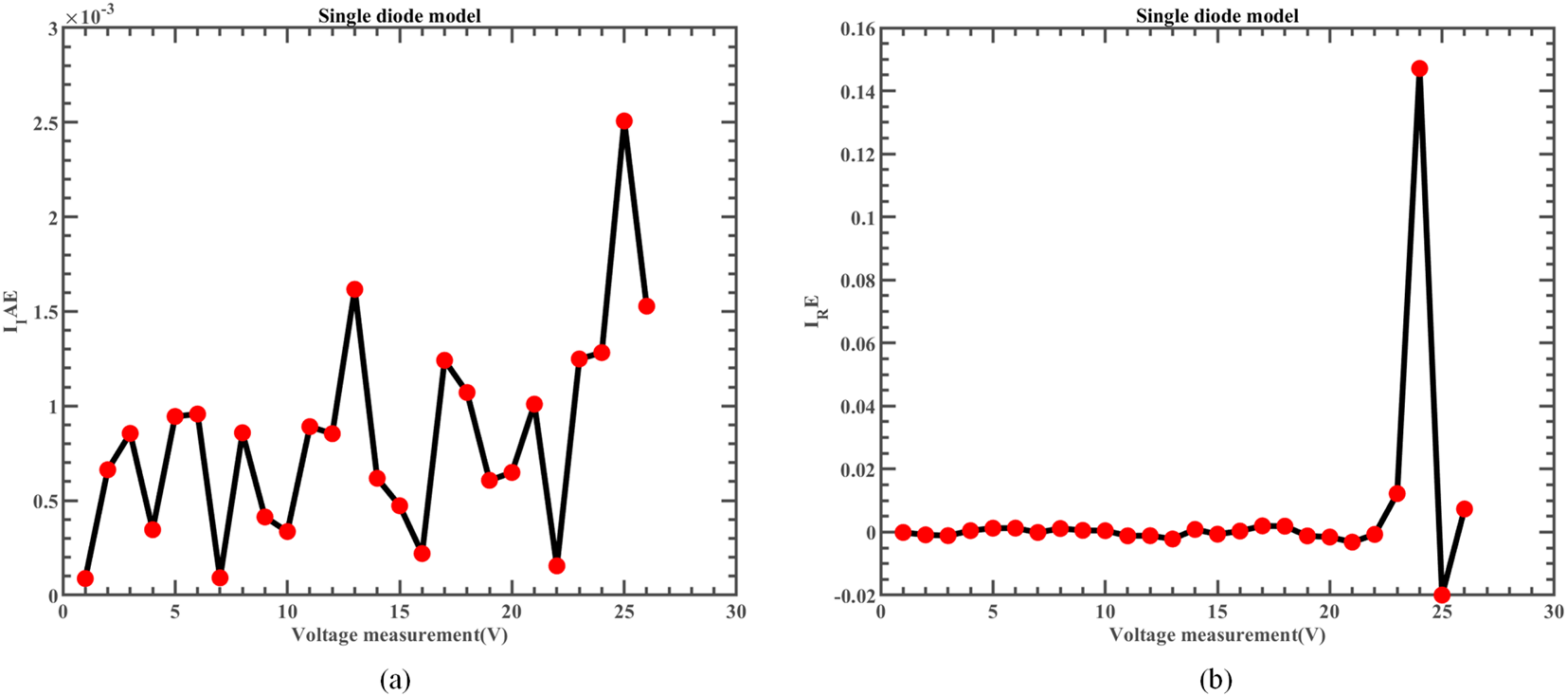
(**a**) The *IAE* value of I with voltage chart of ERINMRIME (**b**) The *RE* value of I with voltage graph of ERINMRIME.

**Figure 16:**
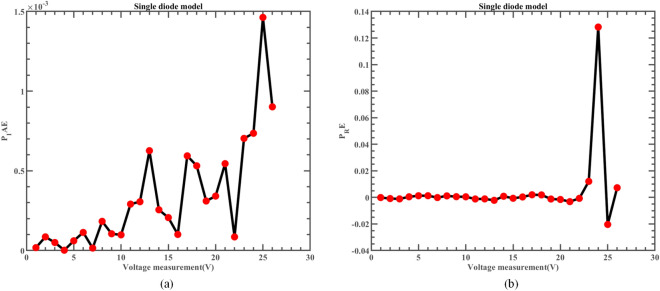
(**a**) The *IAE* value of P with voltage diagram of ERINMRIME (**b**) The *RE* value of P with voltage graph of ERINMRIME.

The fitting comparison of the actual measurement data and simulated data of ERINMRIME on the SDM is shown in Fig. 14. The figure demonstrates that the estimated data produced by ERINMRIME exhibits a high degree of fitting with the actual data. Figure 14 (a) and Fig. 14 (b) display the I and P values of ERINMRIME on the SDM with voltage change, respectively. The specific experimental current and power data are shown in Table A1 (Online Appendix). Figure 15 (a) and Fig. 15 (b) reveal the values of $I_{IAE}$ and $I_{RE}$ that vary with voltage, respectively. The corresponding diagram about $P_{IAE}$ and $P_{RE}$ are shown in Fig. 16. Overall, ERINMRIME exhibits exceptional performance relative to other algorithms due to the perfect combination of two mechanics with RIME. The ERI strategy can help the algorithm avoid local optimization, which allows rime particles to explore new solution space further. NMs mechanism can deeply explore the search for valuable solution regions that have been screened out. The combination of the two characteristics has further boosted the algorithm. The experiments on SDM have also demonstrated the value of adding the above two mechanisms.

### Results on the DDM case

**Figure 17:**
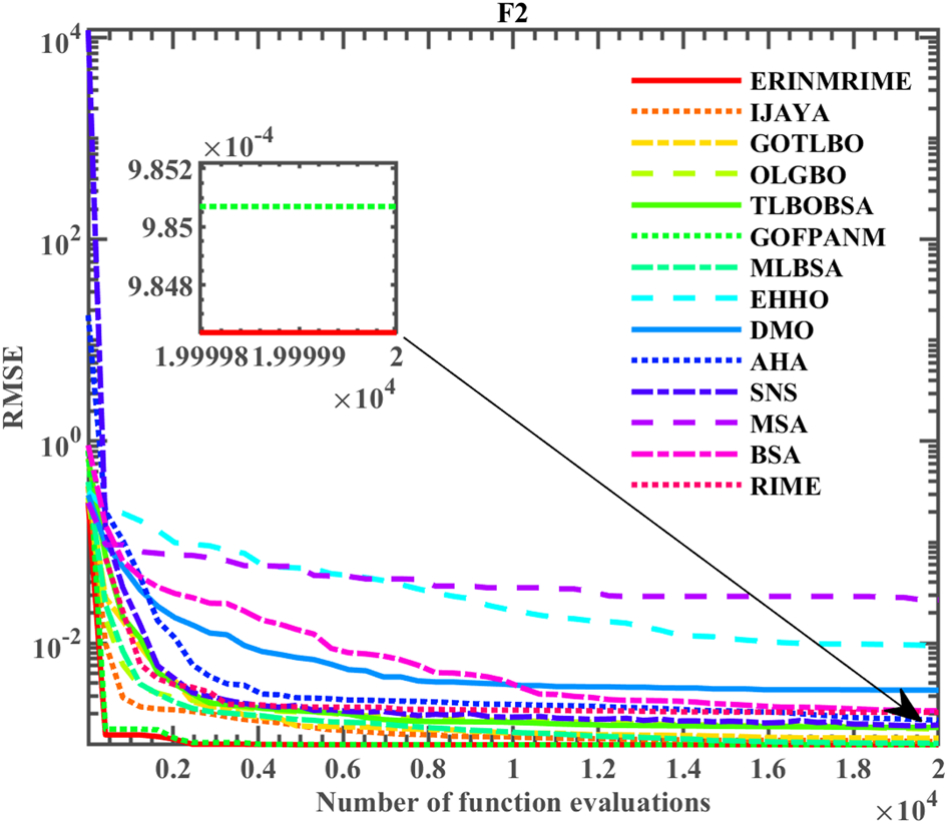
The convergence curve on the DDM.

Likewise, ERINMRIME was also evaluated on the DDM. Table 10 shows the results of the tests. When compared to other algorithms, the table clearly shows that ERINMRIME delivers the lowest *RMSE*, signifying superior performance in the testing phase. Furthermore, its improvement compared to the original RIME algorithm is remarkably significant. In Fig. 17, the specific convergence images of the comparative algorithms are presented, providing a visual representation of ERINMRIME's effectiveness. The graph showcases how ERINMRIME outperforms other algorithms, achieving better convergence and optimization capabilities. The early convergence speed of ERINMRIME is notably faster. While ERINMRIME and GOFPANM exhibit similar trends during the middle and later phases of the algorithm, ERINMRIME outperforms GOFPANM in terms of achieving higher final convergence accuracy. Thus, ERINMRIME exhibits robust accuracy and practicality in the measurement of unknown parameters for the DDM.

**Table 10:** Parameter extraction results of ERINMRIME on DDM.  Significant values are in bold.

Item	$I_{ph} (A)$	$I_{sd1} (\mu A)$	$I_{sd2} (\mu A)$	$R_{s} (\Omega )$	$R_{sh} (\Omega )$	$n_{1}$	$n_{2}$	*RMSE*	Compare
ERINMRIME	0.760781	2.26E−07	0.03674	55.48539	1.451017	7.49E−07	2	**9.82485E−04**	
IJAYA	0.76080	3.03E−07	0.036352	53.73864	1.476351	8.86E−08	1.837405	9.87332E−04	+
GOTLBO	0.760986	2.33E−07	0.036001	55.08775	1.549982	1.61E−07	1.458695	1.03744E−03	+
OLGBO	0.760784	7.92E−07	0.036775	55.58079	1.993179	2.18E−07	1.448185	9.82597E−04	+
TLBOBSA	0.76078	5.12E−07	0.036669	54.97684	1.937493	2.37E−07	1.455315	9.83251E−04	+
GOFPANM	0.760781	2.26E−07	0.03674	55.48544	1.451017	7.49E−07	2	**9.82485E−04**	=
MLBSA	0.760778	4.17E−07	0.036579	54.61718	1.997633	2.66E−07	1.464565	9.83253E−04	+
DMO	0.761086	3.81E−06	0.023889	56.04071	1.036453	1.83E−07	1.581138	1.82426E−03	+
AHA	0.760662	3.57E−07	0.03599	57.16565	1.491229	1.42E−13	1.298283	1.72947E−03	+
SNS	0.760651	3.62E−07	0.035962	58.58556	1.492811	2.45E−10	1.658904	1.54778E−03	+
MSA	0.76958	3.27E−07	0.030209	44.25243	1.5056	6.16E−07	1.739431	2.68674E−02	+
EHHO	0.760332	7.95E−08	0.036141	63.01587	1.464746	2.76E−07	1.499817	1.04230E−03	+
BSA	0.759796	5.18E−07	0.03399	88.65367	1.534499	2.74E−07	1.873659	1.73074E−03	+
RIME	0.760348	3.75E−07	0.035811	65.24357	1.496724	5.5E−08	1.960489	1.07214E−03	+

**Figure 18:**
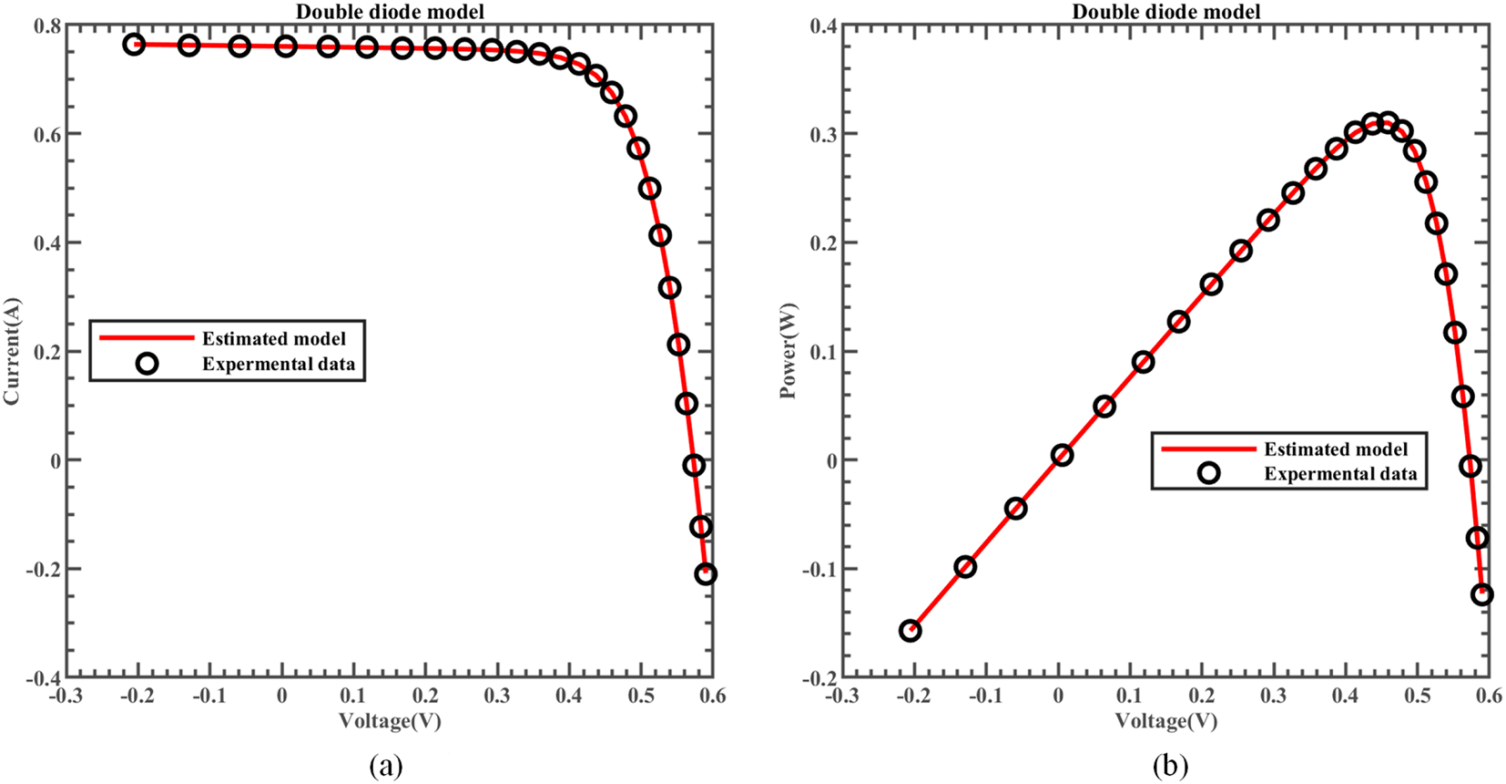
(**a**) The I-V curve of DDM simulated by ERINMRIME (**b**) The P–V graph gained by ERINMRIME.

**Figure 19:**
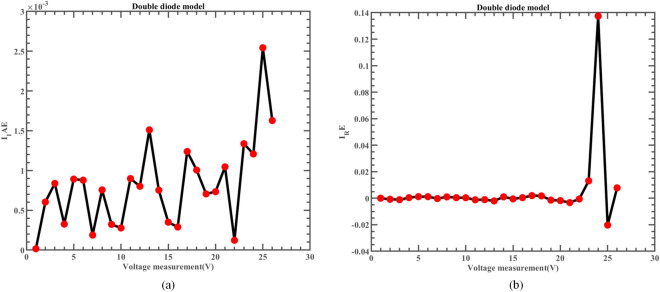
(**a**) The *IAE* value of I with voltage chart of ERINMRIME (**b**) The *RE* value of I with voltage graph of ERINMRIME.

**Figure 20:**
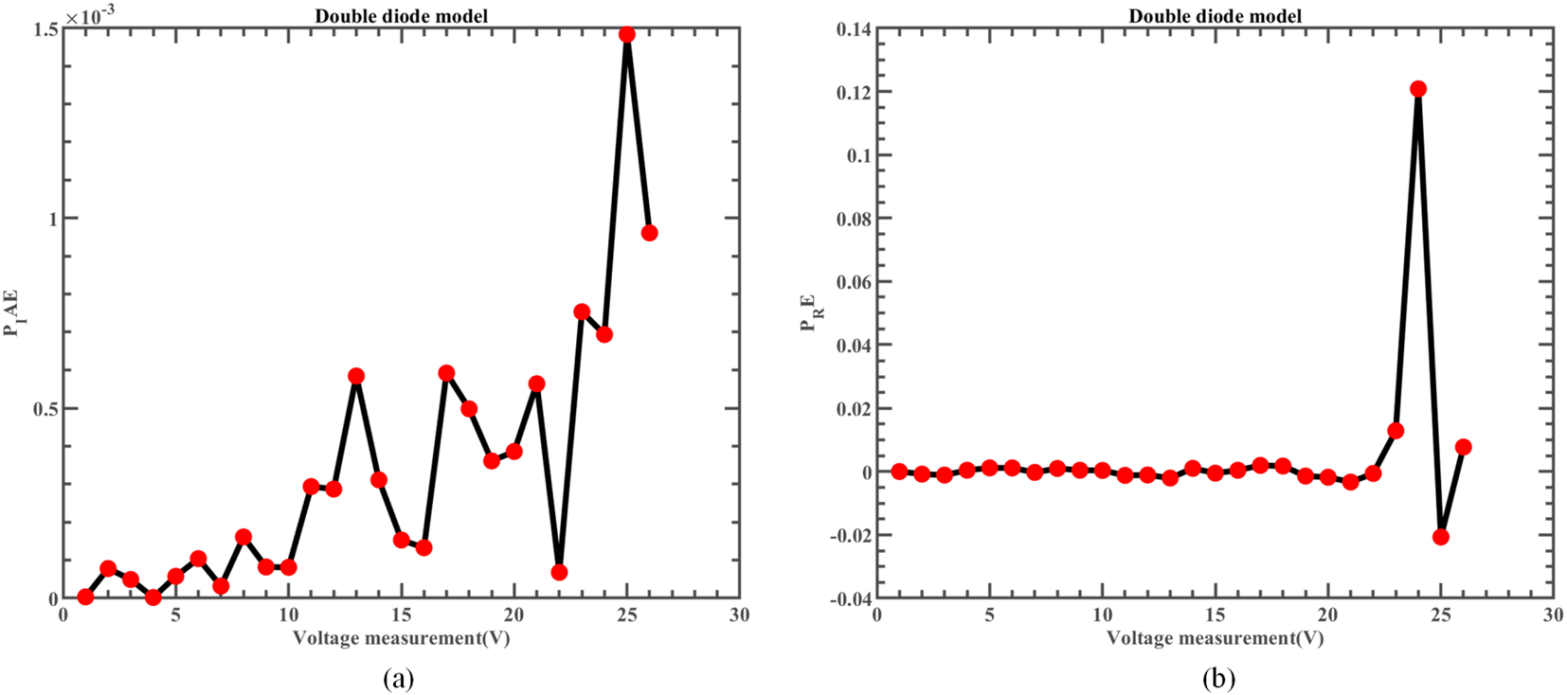
(**a**) The *IAE* value of P with voltage diagram of ERINMRIME (**b**) The *RE* value of P with voltage graph of ERINMRIME.

Figure 18 exhibits the characteristic curve, where the real measured data and simulated data of I-V and P–V from ERINMRIME on the DDM are compared. It is noteworthy that there is a remarkable level of consistency in the fitting of these two forms of data. In Fig. 19, a comparison is presented between the specific data of *IAE* and *RE* of I predicted by ERINMRIME and the corresponding actual data. Detailed data results are presented in Table A2 (Online Appendix). The data that was previously shown makes it clear there is a high degree of agreement in the estimation of unknown data. The ERINMRIME's performance in predicting and estimating unknown values is highly accurate and reliable. The error curve between power and voltage is shown in Fig. 20. To sum up, ERINMRIME has high precision in measuring unknown parameters on DDM.

### Results on the TDM case

**Figure 21:**
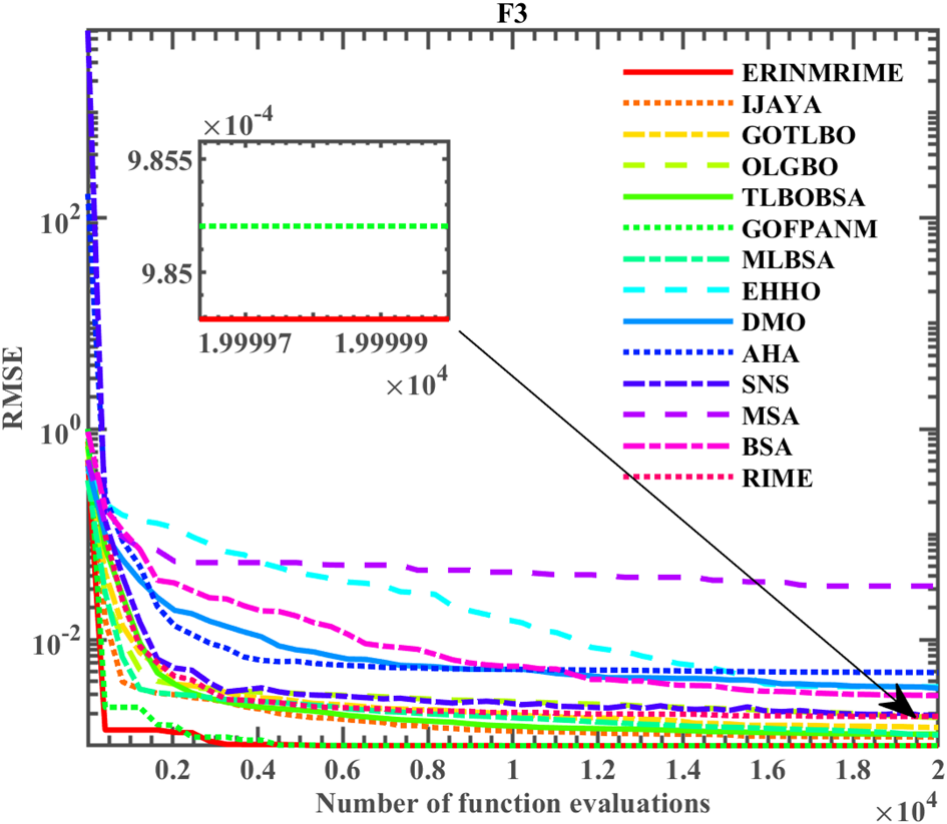
The convergence curve on the TDM.

Similarly, ERINMRIME was applied to the TDM for testing. Table 11 displays the findings of the final comparison. ERINMRIME possesses the optimal *RMSE*. As depicted in Fig. 21, it is evident that the ERINMRIME convergence curve is located around the graph’s bottom. The ERINMRIME has the fastest convergence speed. ERINMRIME has a finite amount of iterations to reach optimal values, demonstrating its strong early optimization capabilities and ability to obtain relatively accurate results quickly. In addition, ERINMRIME owns the smallest *RMSE*, and the converge accuracy is unquestionable. Therefore, ERINMTIME is an effective tool for accurately estimating unknown parameters on TDM.

**Table 11:** Parameter extraction results of ERINMRIME on TDM.  Significant values are in bold.

Item	$I_{ph} (A)$	$I_{sd1} (\mu A)$	$I_{sd2} (\mu A)$	$I_{sd3} (\mu A)$	$R_{s} (\Omega )$	$R_{sh} (\Omega )$	$n_{1}$	$n_{2}$	$n_{3}$	*RMSE*	Compare
ERINMRIME	0.760781	2.28E−07	0.03673	55.4394	1.451861	1.57E−07	2	5.72E−07	2	**9.825E−04**	
IJAYA	0.760659	1E−12	0.036327	53.42182	1.981523	0.00E+00	1.863295	3.26E−07	1.481993147	9.955E−04	+
GOTLBO	0.761278	7.41E−08	0.036672	50.25414	1.842036	2.28E−07	1.452678	3.02E−07	1.868515454	1.029E−03	+
OLGBO	0.760723	3.8E−07	0.036553	55.22338	1.999192	2.51E−08	1.619589	2.56E−07	1.462997168	9.840E−04	+
TLBOBSA	0.760778	2.79E−07	0.036526	54.35388	1.974712	5.25E−09	1.432462	2.75E−07	1.470104145	9.840E−04	+
GOFPANM	0.760781	3.18E−09	0.03674	55.48543	2	2.25E−07	1.451017	7.46E−07	2	**9.825E−04**	**=**
MLBSA	0.760783	5.7E−07	0.036734	55.09995	1.982197	2.11E−07	1.4479	5.26E−08	1.631209212	9.833E−04	+
EHHO	0.760831	4.41E−08	0.036545	52.58998	1.438993	2.42E−07	1.479407	7.18E−08	1.719039247	9.877E−04	+
DMO	0.760598	4.56E−07	0.034997	71.78477	1.508518	2.62E−07	1.862177	1.68E−06	1.410321075	2.332E−03	+
AHA	0.760764	2.7E−07	0.036842	57.12176	1.760071	6.57E−07	1.999592	1.62E−07	1.42734	4.874E−03	+
SNS	0.760704	7.27E−08	0.035981	59.99216	1.395229	4.67E−07	1.615644	2.68E−07	2	1.912E−03	+
MSA	0.758627	2.42E−07	0.033568	36.32192	1.62431	7.71E−07	1.625603	5.12E−07	1.843463	3.222E−02	+
BSA	0.760365	2.27E−07	0.033492	94.99061	1.795295	5.10E−07	1.537888	9.66E−08	1.767952761	1.816E−03	+
RIME	0.759944	3.35E−07	0.035535	76.41091	1.498305	9.47E−08	1.573253	1E−12	1.405128896	1.205E−03	+

**Figure 22:**
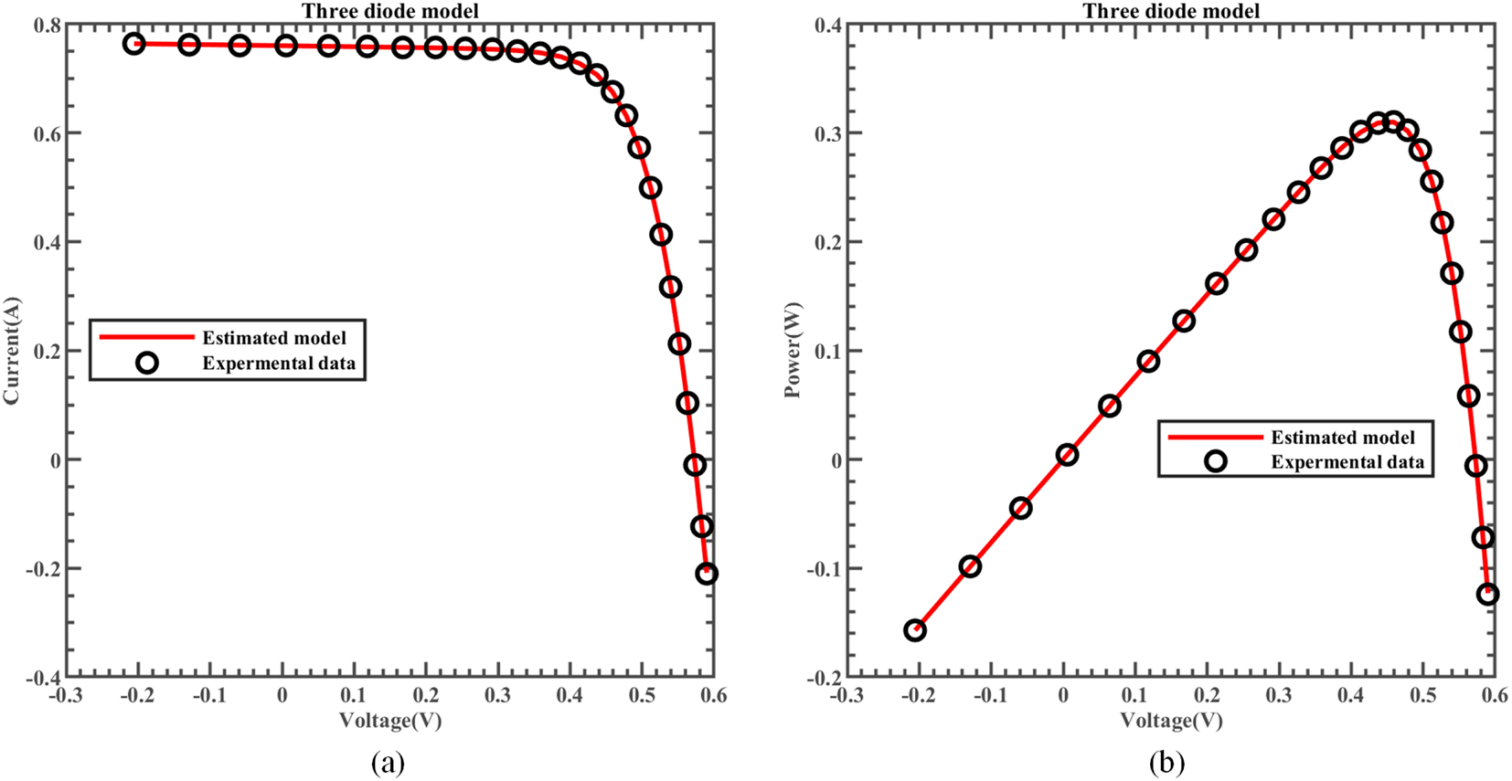
(**a**) The I-V curve of TDM simulated by ERINMRIME (**b**) The P–V graph gained by ERINMRIME.

**Figure 23:**
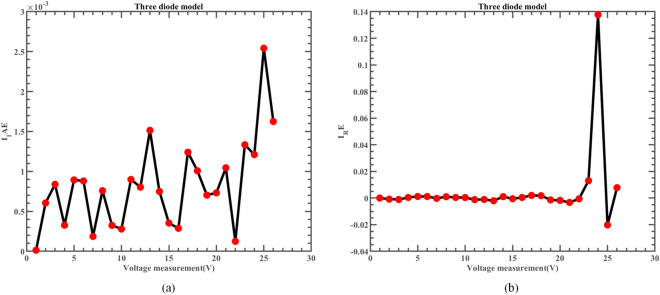
(**a**) The *IAE* value of I with voltage chart of ERINMRIME (**b**) The *RE* value of I with voltage graph of ERINMRIME.

**Figure 24:**
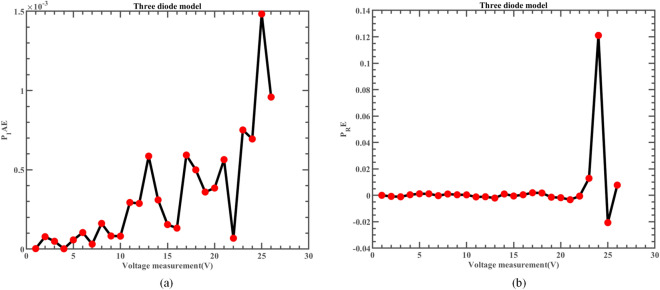
(**a**) The *IAE* value of P with voltage diagram of ERINMRIME (**b**) The *RE* value of P with voltage graph of ERINMRIME.

Likewise, Fig. 22 illustrates the fitting comparison between the detailed estimated data generated by ERINMRIME and the actual data. Based on the images provided, it is evident that the experimental data exhibit a strong and favorable agreement with the simulation model. The simulation model’s curve accurately passes through the central points of the data, showing a high level of accuracy in the fitting process. Table A3 (Online Appendix) contains the comprehensive facts. In addition, the changes of *IAE* and *RE* of ERINMRIME on I and P along with voltage are shown in Figs. 23 and 24. It is noticeable that the relative error of the data measured by ERINMRIME is minimal, indicating a high degree of accuracy and precision in the estimation process. In conclusion, compared to previous approaches, ERINMRIME performs better on the TDM.

### Results on the PV module model cases

**Figure 25:**
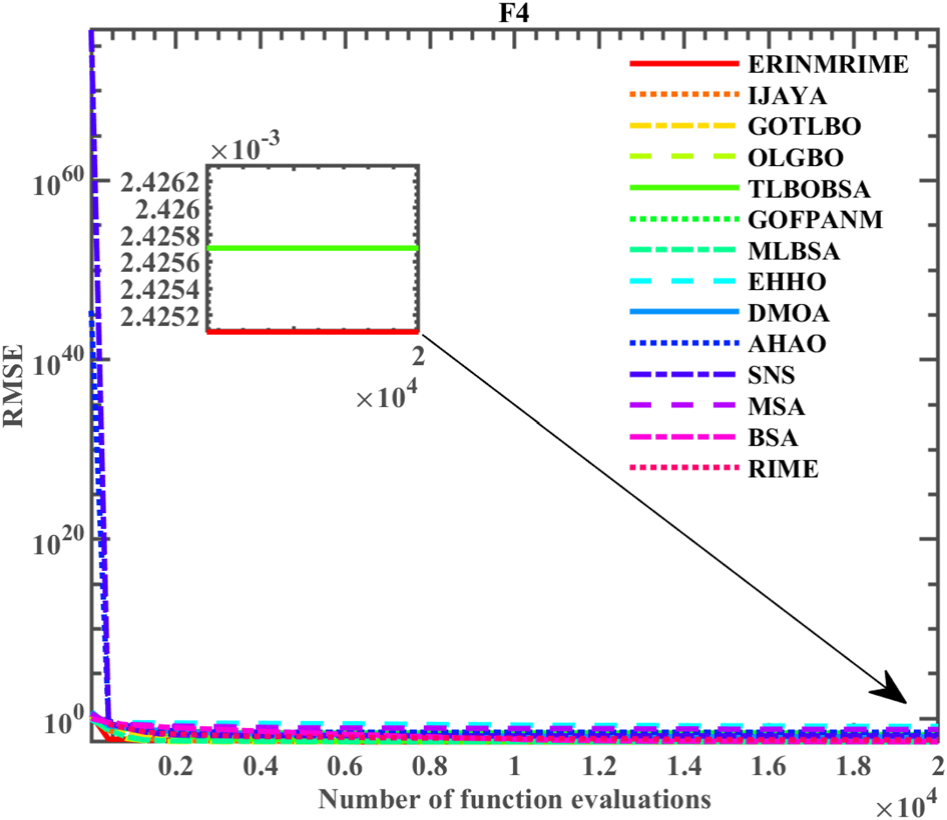
The convergence curve on the PV module model.

Table 12 displays the individual RMSE values of ERINMRIME on the PV module model. Additionally, for a thorough assessment, the related comparison curve with other algorithms is shown in Fig. 25. From the table, it is clear that ERINMRIME's *RMSE* shows a significant improvement compared to RIME. An order of magnitude reduces the RMSE from ERINMRIME. Despite having a similar *RMSE* to the other four common algorithms, the convergence images demonstrate that ERINMRIME achieves the highest final convergence accuracy among all the algorithms. According to the results, ERINMRIME surpasses the other algorithms in terms of optimization precision and accuracy. Meanwhile, the convergence curve of ERINMRIME has a noticeable decline trend, and the convergence speed is good. The optimal solution can be quickly found, and this optimal value is the minimum compared with other algorithms. This demonstrates that ERINMRIME has achieved better results in terms of both convergence speed and accuracy.

**Table 12:** Parameter extraction results of ERINMRIME on PV module model.  Significant values are in bold.

Item	$I_{ph} (A)$	$I_{sd} (\mu A)$	$R_{s} (\Omega )$	$R_{sh} (\Omega )$	*n*	*RMSE*	Compare
ERINMRIME	1.030514	3.48E−06	1.20127	982.0045	48.64289	**2.42507E−03**	
IJAYA	1.030193	3.51E−06	1.200148	1010.683	48.6692	2.42753E−03	+
GOTLBO	1.030487	3.5E−06	1.200535	987.8613	48.66741	2.42515E−03	+
OLGBO	1.030514	3.48E−06	1.201271	981.9834	48.64284	**2.42507E−03**	=
TLBOBSA	1.030514	3.48E−06	1.201271	981.9823	48.64283	**2.42507E−03**	=
GOFPANM	1.030514	3.48E−06	1.201271	981.9823	48.64284	**2.42507E−03**	=
MLBSA	1.030514	3.48E−06	1.201271	981.9884	48.64285	**2.42507E−03**	=
EHHO	1.025774	2.89E−06	1.233255	1906.922	47.92097	3.08629E−03	+
DMO	1.025232	3.04E−05	1.284336	995.6781	53.24275	2.56478E−03	+
AHA	1.030512	3.48E−06	1.201257	982.2828	48.64342	2.96501E−02	+
SNS	1.030515	3.48E−06	1.201394	981.2929	48.63863	1.16719E−02	+
MSA	1.046835	3.88E−06	1.183234	618.1928	49.17334	5.97953E−02	+
BSA	1.030518	3.23E−06	1.211692	958.3982	48.35001	2.44458E−03	+
RIME	1.030073	3.33E−06	1.207456	1016.304	48.46957	2.43827E−03	+

**Figure 26:**
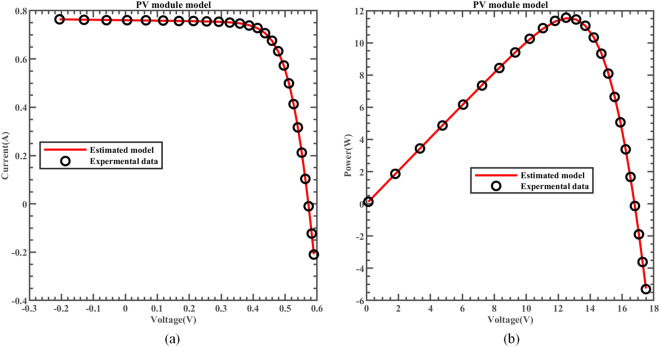
(**a**) The I-V curve of PV simulated by ERINMRIME (**b**) The P–V graph gained by ERINMRIME.

**Figure 27:**
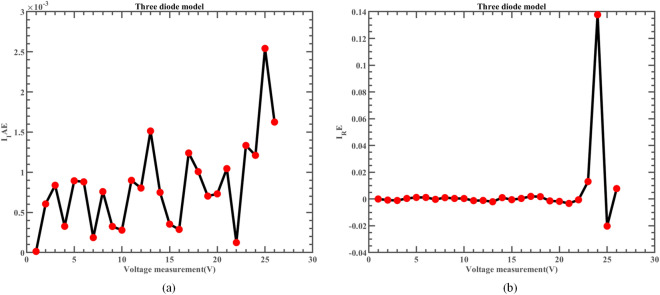
(**a**) The *IAE* value of I with voltage chart of ERINMRIME (**b**) The *RE* value of I with voltage graph of ERINMRIME.

**Figure 28:**
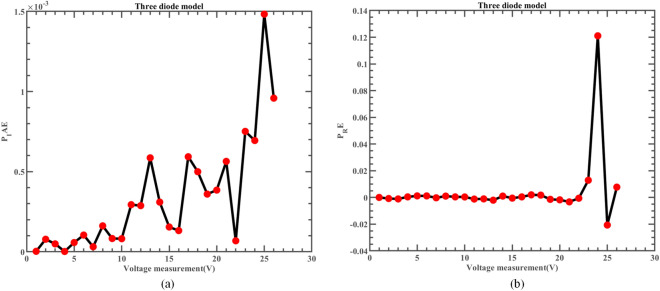
(**a**) The *IAE* value of P with voltage diagram of ERINMRIME (**b**) The *RE* value of P with voltage graph of ERINMRIME.

In addition, this study compares the data estimated by ERINMRIME with the actual data and finally gets 25 groups of specific experimental results, as displayed in Table A4 (Online Appendix). The specific fitting image of the actual and estimated data is shown in Fig. 26. Apparently, the fitting of the two is highly coincident. Correspondingly, Fig. 27 and Fig. 28 illustrate the variations in *IAE* and *RE* for ERINMRIME concerning the parameters I and P at various voltage levels. The findings of the  experiment results show that ERINMRIME can accurately identify PV module model parameters. In conclusion, ERINMRIME performs remarkably well in various scenarios, making it a valuable algorithm for the in-depth exploration of PV model parameter recognition. Its effectiveness and versatility suggest that ERINMRIME is a promising choice for tackling complex PV modeling and optimization challenges.

### Time comparison between ERINMRIME and other algorithms

This study compares the running times of each algorithm on different models, as shown in Table 13. ERINMRIME exhibits comparable running times to other algorithms, but its convergence accuracy is excellent. Although GOFPANM is closest to the convergence accuracy of ERINMRIME, ERINMRIME runs about 300 times faster than GOFPANM. Based on the comprehensive examination of each model’s experimental outcomes provided above, it is determined that ERINMRIME has the fastest convergence speed among them. ERINMRIME can converge quickly with appropriate precision in relatively little time, which benefits from adding an ERI strategy. It enables ERINMRIME to explore the region fully through simple operations to expand its broader exploration without increasing the complexity of the algorithm. Simultaneously, the NMs mechanism also develops local areas in depth with low computational complexity.

**Table 13:** Time comparison between ERINMRIME and various algorithms.

	SDM	DDM	TDM	PV
ERINMRIME	33.56719	33.21615	35.18646	33.07865
IJAYA	32.68698	32.34531	33.00677	32.31667
GOTLBO	33.13854	32.68125	33.55993	32.81042
OLGBO	180.9495	179.6359	184.4547	181.6427
TLBOBSA	32.68594	32.39742	33.42448	32.84271
GOFPANM	7424.262	10,811.01	14,970.83	7588.537
MLBSA	32.06042	31.54167	32.65417	32.31563
EHHO	51.99167	51.15469	52.73281	52.61979
DMO	34.27086	33.38546	54.27083	38.54681
AHA	40.05215	35.57293	50.15639	35.57292
SNS	42.91674	36.47928	55.57293	30.62549
MSA	48.54173	38.43757	56.25448	42.26045
BSA	31.98177	31.41354	32.365102	32.18229
RIME	31.89375	31.43333	32.309937	31.99018

### Results based on the manufacturer’s datasheet

ERINMRIME was used to test the efficacy of SDM and DDM's parameter estimates on three widely used commercial model components, namely single crystal SM55^97^, thin film ST40^98^, and polycrystal KC200GT^99^. The results further demonstrate the practicality and reliability of ERINMRIME in accurately estimating parameters for various PV models, solidifying its potential for real-world applications. The experiments were tested under different irradiance and temperatures, respectively. This comprehensive testing approach ensures that the algorithm can effectively handle the variations in irradiance and temperature, making it reliable for real-world applications in diverse operating conditions. Among them, the best experimental results are indicated in bold. The respective PV model manufacturer supplied each of the three models's data sets.

#### Results for Thin-film SM55 datasheet

The SM55 is a solar cell consisting of 36 PowerMax® single crystal series. The SM55 used in this study is produced by Shell Solar company. Please refer to the literature for specific specifications and parameters^97^.

Table A5 (Online Appendix) shows the specific parameters list of ERINMRIME under different irradiation at 25℃. To thoroughly manifest the accuracy of the ERINMRIME in estimating PV model parameters, this experiment compared ERINMRIME with other seven commonly used classical algorithms (RIME, DSCSE^100^, GOTLBO, IJAYA, EHHO, MLBSA, BSA) at five different irradiation. The specific results are presented in Table 14. ERINMRIME exhibits the lowest *RMSE* in lower irradiation environments and ranks second only to MLBSA in two slightly higher irradiation environments. The results indicate that ERINMRIME exhibits remarkable competitiveness in precisely estimating the parameters of PV models, particularly in low irradiation environments. Table A8 (Online Appendix) indicates the specific parameter settings of the PV models for different temperatures. Similarly, ERINMRIME is compared with other algorithms under the same environmental conditions. The comparison findings are presented in Table 15. As depicted in the chart, the *RMSE* of ERINMRIME is the lowest among all the algorithms. ERINMRIME is less affected by temperature variations. ERINMRIME has a certain temperature tolerance and relatively stable measurement effect.

**Table 14:** *RMSE* for SM55 various irradiance conditions at 25.

Item	*RMSE*				
	200 W/m^2^	400 W/m^2^	600 W/m^2^	800 W/m^2^	1000 W/m^2^
**SDM**					
ERINMRIME	**0.000520541521 **	**0.000707608273 **	**0.000823950530 **	0.000668580109	0.001146215069
RIME	0.001850393417	0.003603214650	0.009799271486	0.012525072955	0.009789888691
DSCSE	0.000521218752	0.001070273347	0.002690384355	0.002548806529	0.004395007384
GOTLBO	0.005278111885	0.006381029606	0.008724529529	0.000658268662	0.002556845481
IJAYA	0.005029446732	0.004765035897	0.007189625687	0.000607771882	0.001843949652
EHHO	0.009206414355	0.011549103756	0.022797244286	0.003477534511	0.006802678806
MLBSA	0.004491956524	0.005698453145	0.006560015285	**0.000577769253 **	**0.000957224353 **
BSA	0.010052275361	0.010891597395	0.016695875024	0.003955715317	0.006337469646
**DDM**					
ERINMRIME	**0.000520541518 **	**0.000599291710 **	**0.000823949402 **	**0.000668579466 **	0.001146214644
RIME	0.013696130659	0.006224646635	0.008322233675	0.003309309629	**0.000936554991 **
DSCSE	0.000566245009	0.000670018097	0.002478143904	0.004159754520	0.003350535294
GOTLBO	0.000683900742	0.002337977953	0.004597227969	0.006930843394	0.007478727788
IJAYA	0.000621476812	0.001817143394	0.002726757865	0.004593508551	0.005571939757
EHHO	0.004164059837	0.005830122630	0.004085320343	0.009212839996	0.026989010587
MLBSA	0.000623905005	0.000798389910	0.000866874790	0.003084008919	0.007848431125
BSA	0.003854136055	0.005200991748	0.008807082610	0.010704004540	0.021477587934

Significant values are in bold.

**Table 15:** *RMSE* for SM55 at various temperatures and 1000 W/m^2^ irradiance.

Item	*RMSE*		
	25 ℃	40 ℃	60 ℃
**SDM**			
ERINMRIME	**0.001146215026 **	**0.003788814684 **	**0.003780388110 **
RIME	0.028271791658	0.012875227497	0.005407329612
DSCSE	0.005667164612	0.004019095248	0.003780401822
GOTLBO	0.007685764072	0.006443556599	0.004296128351
IJAYA	0.007394004709	0.004781433599	0.003971632213
EHHO	0.012411250164	0.011366821143	0.007764462446
MLBSA	0.003249552353	0.005984318560	0.003800324217
BSA	0.012586238996	0.010637931604	0.006737203346
**DDM**			
ERINMRIME	**0.001146214644 **	**0.003788814655 **	**0.003780388066 **
RIME	0.023560612106	0.009928594211	0.010251314506
DSCSE	0.006963933706	0.004113609290	0.003783226603
GOTLBO	0.007487426866	0.008184120923	0.004249811491
IJAYA	0.005697600940	0.004984418141	0.004249314077
EHHO	0.017896838464	0.025613516230	0.006382333338
MLBSA	0.007893411238	0.005621252276	0.004128671264
BSA	0.014137640863	0.011672675947	0.006692389046

Significant values are in bold.

**Figure 29:**
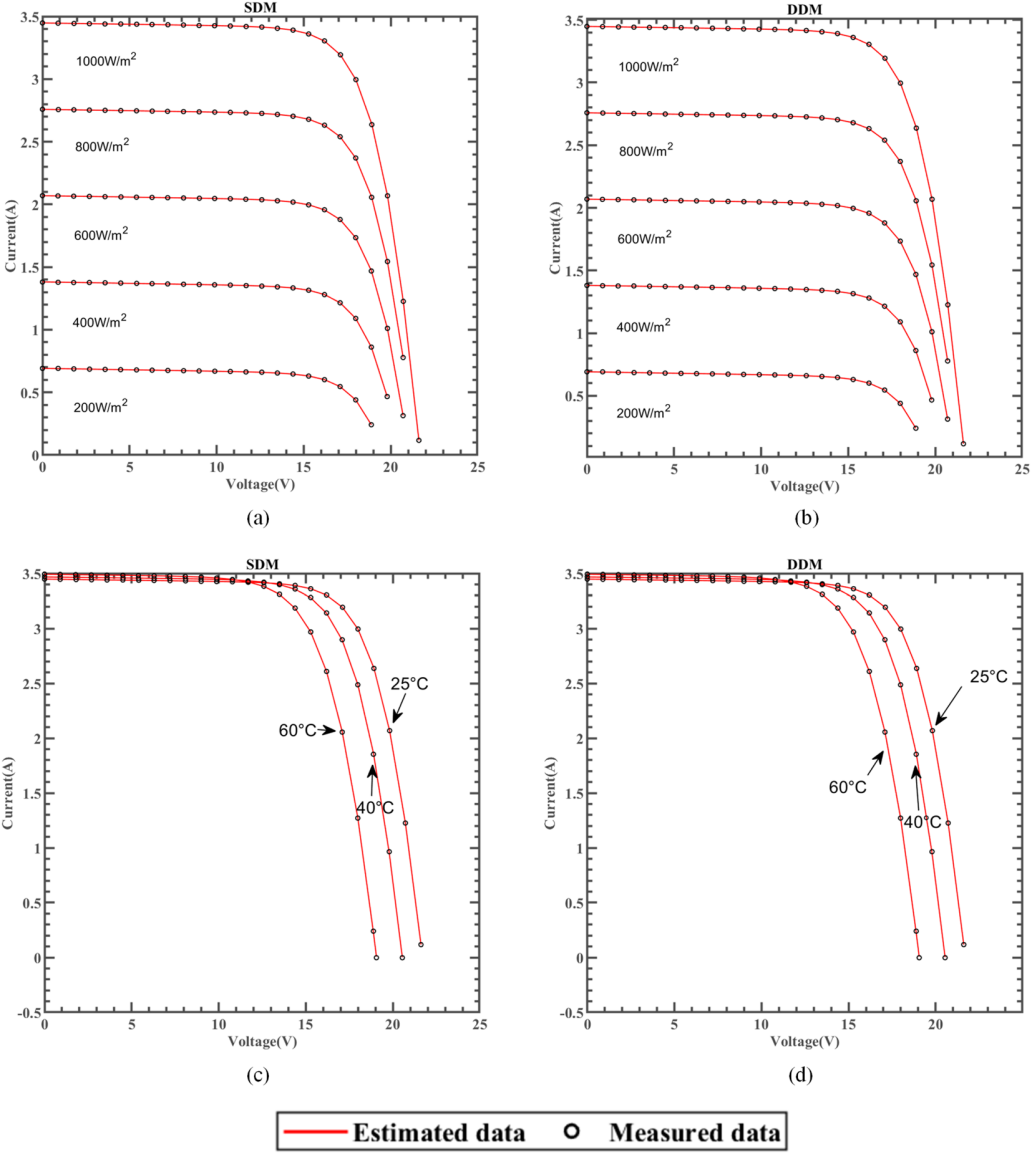
(**a**, **b**) The I-V curves of SDM and DDM for SM55 simulated by ERINMRIME at 25 ℃ under various irradiances (**c**, **d**) The I-V graph of SDM and DDM acquired by ERINMRIME at various temperatures in the irradiance of 1000 W/m^2^.

Figure 29 (c, d) displays the specific current and voltage values of the ERINMRIME based on the SDM or DDM battery model on SM55 at different temperatures (25 ℃, 40 ℃, 60 ℃) under the condition of the fixed variable 1000 W/m^2^. Noticeably, the estimation of series parameters of the SM55 by ERINMRIME is consistent with the height of the actual measured data. Similarly, in Fig. 29 (a, b), when different irradiances are tested at a fixed variable of 25 ℃, it can be distinctly found that the relative transformation of irradiation almost does not influence the measurement of the algorithm. To sum up, the estimation ability of ERINMRIME in the actual PV models has been greatly affirmed. It is a valuable algorithm worthy of further exploration in the measurement of PV model parameters.

#### Results for Thin-film ST40 datasheet

 Composed of copper indium selenide (CIS) formed in succession by solar cells, ST40 is a monolithic structure.  The ST40 used in this study was produced by Shell Solar. Please refer to the literature for specific specifications and parameters^98^.

**Figure 30:**
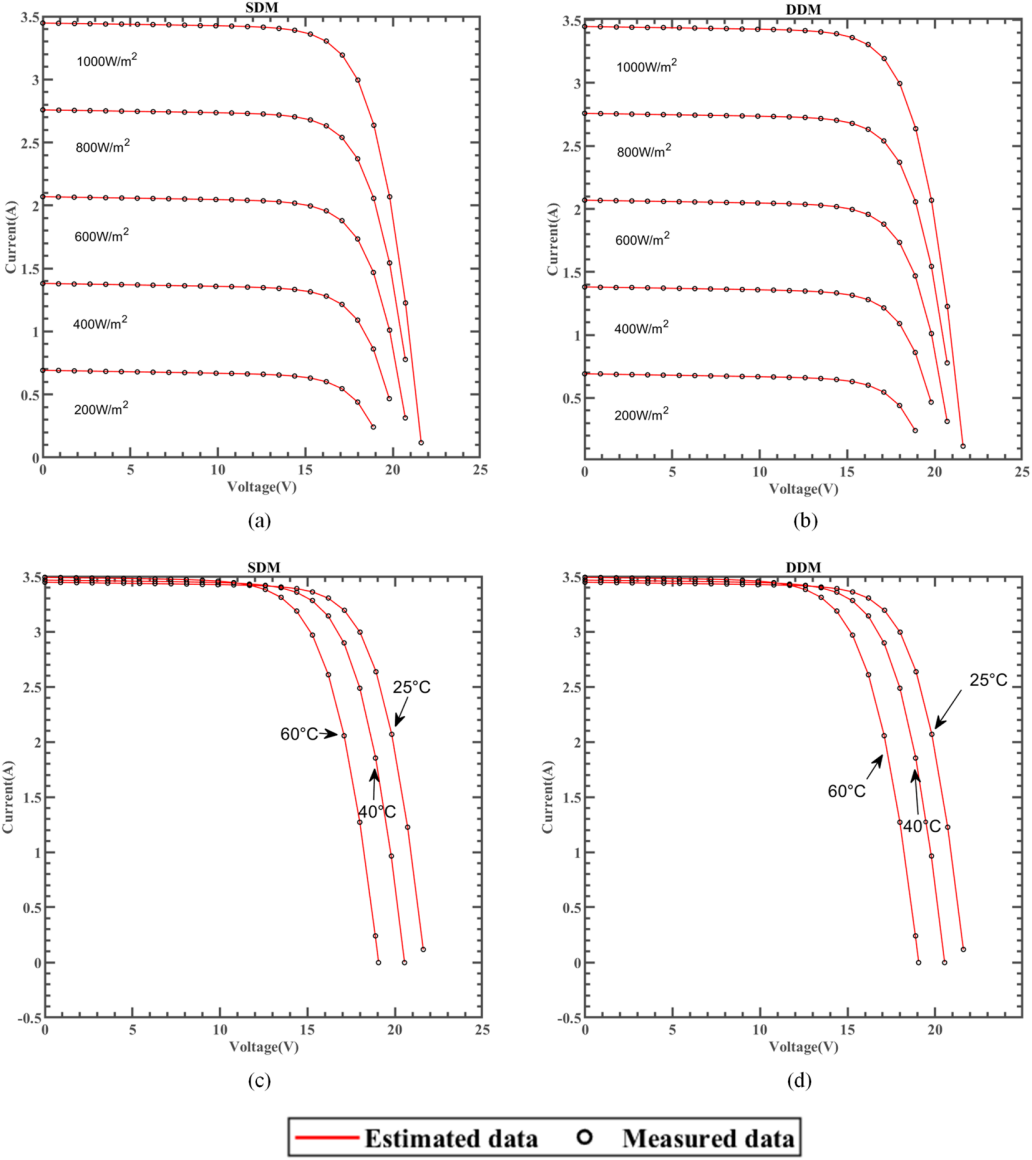
(**a**, **b**) The I-V curves of SDM and DDM for ST40 simulated by ERINMRIME at 25 ℃ under varied irradiances (**c**, **d**) The I-V graph of SDM and DDM obtained by ERINMRIME at various temperatures in the irradiance of 1000 W/m^2^.

Table A6 (Online Appendix) displays the list of specific parameters of different irradiation of ERINMRIME at 25 ℃. Figure 30 (a, b) shows the fitting images of the measured data and estimated data acquired by ERINMRIME at ST40 consisting of SDM or DDM. ERINMRIME's accuracy is guaranteed under a variety of irradiation conditions. There will be no relatively large change in accuracy with the irradiation change. This study compared ERINMRIME and other algorithms based on the *RMSE* assessment criterion in order to better illustrate the estimation algorithm's accuracy. As shown in Table 16, the *RMSE* of ERINMRIME was always the smallest. The above phenomenon deeply proved that ERINMRIME is a valuable algorithm. It can be applied well to practical PV models.

**Table 16:** *RMSE* at 25 °C for various ST40 irradiation conditions.

Item	*RMSE*				
	200 W/m^2^	400 W/m^2^	600 W/m^2^	800 W/m^2^	1000 W/m^2^
**SDM**					
ERINMRIME	**0.000477200794 **	**0.000630724727 **	**0.000674035939 **	**0.000773905214 **	**0.000734098527 **
RIME	0.001378237434	0.003034474245	0.003882856920	0.006325711542	0.004436648337
DSCSE	0.000477311656	0.000751594536	0.000780510732	0.001353166081	0.001247134172
GOTLBO	0.000597489423	0.001996929214	0.002521561884	0.005508556685	0.005366236974
IJAYA	0.000537152709	0.000717432668	0.001312434093	0.005056216149	0.003594811748
EHHO	0.003443411121	0.004731924715	0.006017680665	0.007844084632	0.011706146831
MLBSA	0.000478500634	0.001254456560	0.001441377396	0.003294462981	0.003878239069
BSA	0.001787166928	0.003921856585	0.005402578196	0.007861487660	0.010265855040
**DDM**					
ERINMRIME	**0.000454478521 **	**0.000630724727 **	**0.000674035939 **	**0.000773905842 **	**0.000734099454 **
RIME	0.001160195985	0.003987383968	0.005071554507	0.005972987766	0.008405972693
DSCSE	0.000468297426	0.000638686845	0.000690226159	0.000817499672	0.000734972819
GOTLBO	0.000671054675	0.002736125511	0.003925716054	0.005245153949	0.005262762862
IJAYA	0.000549122970	0.001827428077	0.000899881255	0.004854223462	0.004947178271
EHHO	0.003312383069	0.005730118672	0.006777552185	0.008617012823	0.011973131319
MLBSA	0.000468634411	0.000972465679	0.000784598227	0.002449621397	0.002746782136
BSA	0.002567750683	0.002623231448	0.005573303964	0.007305934300	0.007043568002

Significant values are in bold.

In addition, this study examined the precise results of the PV parameters measured by ERINMRIME at the fixed irradiation condition of 1000 W/m^2^ under four different temperatures to confirm the influence of temperature on the measured data. Table 17 displays the specific values. Fig. 30 (c, d) displays the fitting curve between the observed values and the corresponding specified current and voltage values. From the tables and images, it is evident that the estimated data of ERINMRIME shows a high level of fit with real data. To further validate the competitiveness of ERINMRIME, this study conducted a comparison with other classic algorithms at the four different temperatures previously indicated, confirming its superiority. Ultimately, the algorithm demonstrated prominent advantages over the other methods in accurately estimating the PV models in various temperature conditions.

**Table 17:** *RMSE* for ST40 at different temperatures and 1000 W/m^2^ irradiance.

Item	*RMSE*			
	25 ℃	40 ℃	50 ℃	70 ℃
**SDM**				
ERINMRIME	**0.000734099407 **	**0.001321414138 **	**0.001823260128 **	**0.000777718049 **
RIME	0.003957726074	0.002998751916	0.003635321790	0.000807566242
DSCSE	0.001390773027	0.001321458491	**0.001823260066 **	**0.000777718042 **
GOTLBO	0.003309967788	0.003922455403	0.002623107114	0.000781637193
IJAYA	0.005189050921	0.003610406879	0.002289230087	0.000778199466
EHHO	0.007050195115	0.009969989602	0.005835524024	0.005282492377
MLBSA	0.003575709828	0.002317575518	0.001841203630	0.000777718043
BSA	0.007889446568	0.006981800203	0.004161814492	0.000970681213
**DDM**				
ERINMRIME	**0.000734098527 **	**0.001321413734 **	**0.001545938824 **	**0.000777718042 **
RIME	0.004288262125	0.003659762600	0.002466291837	0.001015166674
DSCSE	0.000734897420	0.001330024844	0.001698633467	**0.000777718043 **
GOTLBO	0.004985794237	0.003464395011	0.003209905138	0.000821686572
IJAYA	0.003826714286	0.002860470517	0.002017390633	0.000784833151
EHHO	0.013368881359	0.009440846505	0.005968279482	0.004347543697
MLBSA	0.003211478993	0.002432410339	0.001758711822	0.000777862597
BSA	0.006004689729	0.006361762643	0.003863446534	0.001851039491

Significant values are in bold.

#### Results for Thin-film KC200GT datasheet

An efficient polycrystalline silicon PV model is the KC200GT. The KC200GT used in this study is produced by KYOCERA Corporation. Please refer to the literature for specific specifications and parameters^99^.

**Figure 31:**
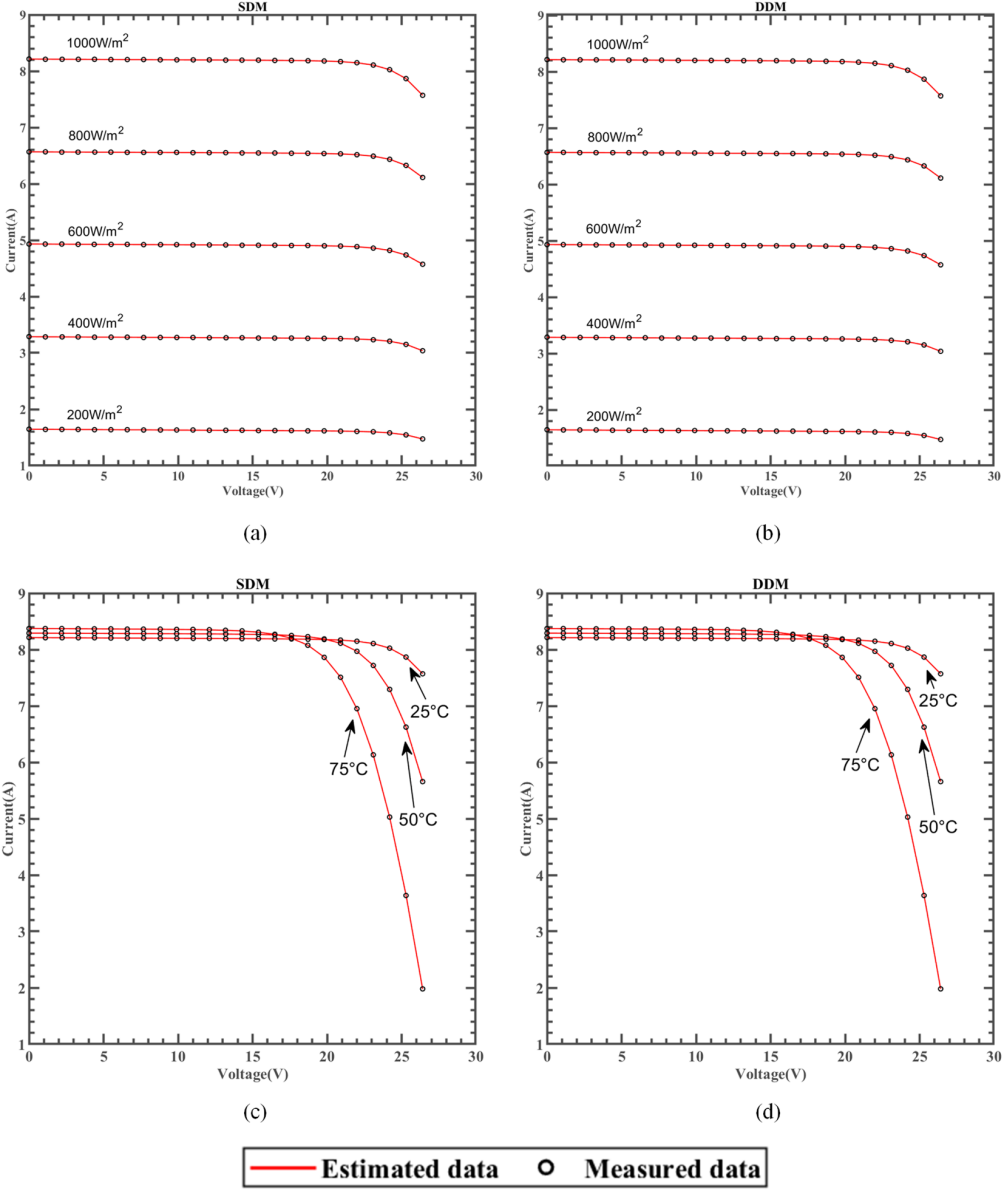
(**a**, **b**) The I-V curves of SDM and DDM for KC200GT simulated by ERINMRIME at 25 ℃ under diverse irradiances (**c**, **d**) The I-V graph of SDM and DDM gained by ERINMRIME at various temperatures in the irradiance of 1000 W/m^2^.

To further verify the accuracy of measurements generated by ERINMRIME on the PV models. This study conducted deep testing on the commercial model KC200GT. Table A7 (Online Appendix) reveals the specific parameter list of ERINMRIME under different irradiations at 25 ℃. Figure 31 (a, b) shows the fitted images consisting of the actual measurement values of PV models composed of SDM or DDM of KC200GT corresponding to measured data of ERINMRIME. It can be distinctly seen that the error between the two kinds of data is very small, which can guarantee the corresponding relationship of accuracy. Moreover, the measurement results of ERINMRIME will not have relatively large precision changes with the changes of irradiation. To further demonstrate the improvement of the estimation data of the algorithm, this study compared ERINMRIME with other algorithms on the evaluation standard of *RMSE*. As shown in Table 18, the *RMSE* of ERINMRIME is always the smallest among them. ERINMRIME is an algorithm with improvement significance, strong competitiveness and optimization ability.

**Table 18:** *RMSE* for KC200GT different irradiance conditions at 25 °C.

Item	*RMSE*				
	200 W/m^2^	400 W/m^2^	600 W/m^2^	800 W/m^2^	1000 W/m^2^
**SDM**					
ERINMRIME	**0.001423293376 **	**0.001336437271 **	**0.001348978626 **	**0.001298424381 **	**0.001206083458 **
RIME	0.002618170807	0.003265205387	0.002807315036	0.003126907557	0.003045015849
DSCSE	0.001442623263	0.001388069376	0.001357883646	0.001429229345	0.001716702692
GOTLBO	0.001451700238	0.001410115315	0.001381836154	0.001485999147	0.001884717636
IJAYA	0.001459319336	0.001382503821	0.001373965172	0.001479896760	0.001891262625
EHHO	0.002330309030	0.003023824181	0.003075731512	0.003556568236	0.004369895424
MLBSA	0.001443887214	0.001402837364	0.001368256408	0.001477195327	0.001828433362
BSA	0.004372724641	0.006558115191	0.003634737148	0.006659252865	0.009709207943
**DDM**					
ERINMRIME	**0.001423293376 **	**0.001336437270 **	**0.001348978625 **	**0.001298424379 **	**0.001205869244 **
RIME	0.002146985490	0.002682283056	0.002892438871	0.001850891851	0.003382581198
DSCSE	0.001438613769	0.001455801391	0.001368410789	0.001466216314	0.001815886926
GOTLBO	0.001452051505	0.001433772964	0.001387878959	0.001500037037	0.001752431113
IJAYA	0.001455250234	0.001413567213	0.001372279566	0.001475688900	0.001857962342
EHHO	0.004980305325	0.003821270693	0.002874883509	0.003473339599	0.004876841805
MLBSA	0.001442227919	0.001398875083	0.001350565022	0.001488188870	0.001289467446
BSA	0.004833723593	0.006870409191	0.007342237964	0.004448645353	0.006404921051

Significant values are in bold.

Furthermore, this study tested the algorithm results under fixed irradiation conditions 1000 W/m^2^ at three different temperatures in order to thoroughly verify the impact of temperature on the recorded data. The corresponding measured values can be found in Table A10 (Online Appendix). Fig. 31 (c, d) displays the fitting curve between the observed values and the particular current and voltage values. As evident from the figure, the estimated data obtained by the algorithm exhibit a strong degree of conformity with the actual data. For proving the unique advantages of ERINMRIME and the relative competitiveness of optimization ability, ERINMRIME was compared with other commonly used traditional algorithms under the above three different temperatures. The eventual comparison outcomes are displayed in Table 19. Upon inspection of the bolded data, the ERINMRIME exhibits the smallest *RMSE* values. In conclusion, ERINMRIME can achieve excellent accuracy in the actual PV models under a variety of temperatures and irradiance circumstances.

**Table 19:** *RMSE* for KC200GT at different temperatures and 1000 W/m^2^ irradiance.

Item	*RMSE*		
	25 ℃	40 ℃	50 ℃
**SDM**			
ERINMRIME	**0.001206083469 **	**0.001446768744 **	**0.002794079636 **
RIME	0.004874063054	0.007526467766	0.007552081526
DSCSE	0.001675458795	0.005440257948	0.003363997546
GOTLBO	0.001855539629	0.006312139159	0.010261130714
IJAYA	0.001887115158	0.005980133511	0.003810441942
EHHO	0.004113661859	0.013443571114	0.025975947065
MLBSA	0.001552895953	0.007434040755	0.006843565633
BSA	0.010944383711	0.017688406840	0.033408284362
**DDM**			
ERINMRIME	**0.001205869259 **	**0.001439629259 **	**0.002053404931 **
RIME	0.002744165376	0.007231053413	0.009015489293
DSCSE	0.001765699305	0.003889759230	0.006285840921
GOTLBO	0.001855539629	0.006312139159	0.010261130714
IJAYA	0.001907498606	0.006260936690	0.002982478045
EHHO	0.004431930567	0.017522687150	0.012648848843
MLBSA	0.001913540215	0.005690079986	0.007343715900
BSA	0.009223035176	0.016857687882	0.026641886201

Significant values are in bold.

## Discussion on the results

For the sake of solving the problem of RIME being susceptible to getting trapped in local optima during later iterations, this study introduces the ERI strategy. The unstable form of rime particles deeply interacts with the environment, making rime particles wander more uniformly in the global space of the solution. This strategy facilitates further interaction between the newly generated rime particles and the environment, thereby enhancing the algorithm's capacity to search the solution space and stay out of local optima. In order to balance between exploration and exploitation and to explore the selected valuable local solution space in greater depth, the NMs mechanism is introduced in this study to improve the local search capability of the algorithm. NMs mechanism can optimize the optimal solution, improving its quality. Combining these two mechanisms optimizes RIME, leading to a highly effective and efficient algorithm known as ERINMRIME. The incorporation of the ERI strategy and NMs mechanism has garnered positive feedback and demonstrated exceptional performance in the parameter extraction of PV models.

To showcase ERINMRIME’s efficacy on PV models, this study carried out experiments on various models. ERINMRIME was compared against other advanced algorithms, namely OLGBO, TLBOBSA, IJAYA, GOTLBO, GOFPANM, MLBSA, EHHO, BSA, and RIME, to comprehensively assess its performance and superiority in parameter extraction and optimization tasks. ERI strategy enables rime particles to further communicate with the environment, making the newly generated particles more closely integrated with the environment and climate. The incorporation of the ERI in the optimization of RIME ensures the avoidance of local optima and enhancement of the global search capability of the ERINMRIME. The ERI strategy facilitates thorough exploration of the solution space, while the NMs mechanism iteratively explores the vicinity of promising solutions, enabling ERINMRIME to develop and refine locally. The synergistic combination of these two mechanisms complements each other, resulting in a substantial improvement in the optimization capabilities of ERINMRIME, taking its performance to a higher level. The final experimental results indicate that ERINMRIME is a robust optimization algorithm. In addition, to verify the practicality of the ERINMRIME in practical PV commercial models, this study used three real datasets SM55, ST40, and KC200GT extracted from the manufacturer's dataset to test the PV models respectively. The simulated values obtained through the fitting process are compared with the corresponding real values. The model parameters estimated by ERINMRIME showed a highly fitting state with the actual data. To further confirm the optimization value of this algorithm, this study evaluated it against other traditionalal algorithms for *RMSE* under various temperatures and irradiation levels. ERINMRIME always obtained a lower *RMSE* value. In conclusion, the ERINMRIME proposed in this study has exhibited remarkable competitiveness in identifying unknown parameters in PV models. ERINMRIME is an algorithm worth exploring further for its practical value.

## Conclusions and future directions

The present study introduces an enhanced version of the RIME, referred to as ERINMRIME, that integrates the ERI strategy with the NMs mechanism. The ERINMRIME incorporates a random interaction strategy of the environment, which facilitates thorough interactions between the newly generated RIME particles and the environment. This enhances ERINMRIME's ability to search globally, enabling it to avoid local optima. The ERINMRIME's capacity for local exploitation can be enhanced by the NMs mechanism. The two mechanisms complement each other and finally raises the algorithm's overall performance. To verify the ERINMRIME's performance, this study conducted experiments on PV models. Comparing the findings to the original RIME algorithm, there are noticeable performance gains. Specifically, ERINMRIME achieved performance gains of 46.23%, 59.32%, 61.49%, and 23.95% for SDM, DDM, TDM, and the PV module model, respectively, showcasing its remarkable capabilities in optimizing and extracting parameters for various PV models. In addition, the algorithm was also validated on three commercial models. In the end, the experimental findings show that ERINMRIME performs remarkably well when it comes to PV model parameter extraction.

While ERINMRIME has demonstrated satisfactory performance in parameter optimization for PV models, it may not be as effective in tasks such as object tracking, energy optimization, and multi-objective optimization. In future work, other optimization problems and objectives should be considered. Additionally, addressing multi-objective problems is a hot topic, but it requires balancing and optimizing multiple conflicting objective functions to obtain a set of optimal solutions, which differs from single-objective optimization. Therefore, developing a multi-objective version of ERINMRIME will be a future direction of research.

### Supplementary Information

Supplementary Information.

## Data Availability

All of the public data used in this study are available for download through https://github.com/sanvsdv12556/Photovoltaic-model-dataset?tab=readme-ov-file#photovoltaic-model-dataset.
