# Supplementary material for: Environment random interaction of rime optimization with Nelder-Mead simplex for parameter estimation of photovoltaic models
Source: Sci Rep. 2024 Jul 8;14:15701. doi: 10.1038/s41598-024-65292-x (PMC11231246; doi:10.1038/s41598-024-65292-x)
Supplement: Supplementary file 1 — Supplementary Information. [file 41598_2024_65292_MOESM1_ESM.docx]

# Appendix

**Algorithm A1**：Pseudo-code of ERINMRIME

1. Parameter *N*, *MaxIt*, *t*, *dim*, *w*;

2. **Initialize** a set of rime individuals;

3. $R_{best}$is the best rime individual;

4. **While** (*t* $\leq$ *MaxIt*)

5. **for** $i$= 1: *N*

6*.* **for** *j* = 1: *dim*

7*.* Execute the soft rime strategy according to **Eq. (14) - Eq. (17)**;

8. Execute the hard-rime puncture mechanism according to **Eq. (18)**;

9. **End for**

10. **End for**

11. **for** $i$= 1: *N*

12. Check the boundary conditions of the newly created rime;

13. Follow **Eq. (19) - Eq. (20)** to implement the PGSM;

14. **End for**

15. **for** $i$= 1: *N*

16. Execute ERI strategy according to **Eq. (21) - Eq. (25)**;

17. **End for**

18. Follow **Eq. (26) - Eq. (31)** to implement the NMs mechanism;

19.;

20. **End While**

21. Output the best solution $R_{best}$;

**Table A1**. *IAE* values of ERINMRIME on SDM

| Item | Measured data | | Current data simulated by ERINMRIME | | Power data simulated by ERINMRIME | |
| --- | --- | --- | --- | --- | --- | --- |
|  | $V(V)$ | $I(A)$ | $I_{S}(A)$ | $IAE_{I}(A)$ | $P_{S}(W)$ | $IAE_{P}(W)$ |
| 1 | -0.2057 | 0.764 | 0.764088 | 8.78E-05 | -0.15717 | 1.81E-05 |
| 2 | -0.1291 | 0.762 | 0.762663 | 0.000663 | -0.09846 | 8.56E-05 |
| 3 | -0.0588 | 0.7605 | 0.761355 | 0.000855 | -0.04477 | 5.03E-05 |
| 4 | 0.0057 | 0.7605 | 0.760154 | 0.000346 | 0.004333 | 1.97E-06 |
| 5 | 0.0646 | 0.76 | 0.759055 | 0.000945 | 0.049035 | 6.1E-05 |
| 6 | 0.1185 | 0.759 | 0.758042 | 0.000958 | 0.089828 | 0.000113 |
| 7 | 0.1678 | 0.757 | 0.757092 | 9.16E-05 | 0.12704 | 1.54E-05 |
| 8 | 0.2132 | 0.757 | 0.756141 | 0.000859 | 0.161209 | 0.000183 |
| 9 | 0.2545 | 0.7555 | 0.755087 | 0.000413 | 0.19217 | 0.000105 |
| 10 | 0.2924 | 0.754 | 0.753664 | 0.000336 | 0.220371 | 9.83E-05 |
| 11 | 0.3269 | 0.7505 | 0.751391 | 0.000891 | 0.24563 | 0.000291 |
| 12 | 0.3585 | 0.7465 | 0.747354 | 0.000854 | 0.267926 | 0.000306 |
| 13 | 0.3873 | 0.7385 | 0.740117 | 0.001617 | 0.286647 | 0.000626 |
| 14 | 0.4137 | 0.728 | 0.727382 | 0.000618 | 0.300918 | 0.000256 |
| 15 | 0.4373 | 0.7065 | 0.706973 | 0.000473 | 0.309159 | 0.000207 |
| 16 | 0.459 | 0.6755 | 0.67528 | 0.00022 | 0.309954 | 0.000101 |
| 17 | 0.4784 | 0.632 | 0.630758 | 0.001242 | 0.301755 | 0.000594 |
| 18 | 0.496 | 0.573 | 0.571928 | 0.001072 | 0.283676 | 0.000532 |
| 19 | 0.5119 | 0.499 | 0.499607 | 0.000607 | 0.255749 | 0.000311 |
| 20 | 0.5265 | 0.413 | 0.413649 | 0.000649 | 0.217786 | 0.000342 |
| 21 | 0.5398 | 0.3165 | 0.31751 | 0.00101 | 0.171392 | 0.000545 |
| 22 | 0.5521 | 0.212 | 0.212155 | 0.000155 | 0.117131 | 8.54E-05 |
| 23 | 0.5633 | 0.1035 | 0.102251 | 0.001249 | 0.057598 | 0.000703 |
| 24 | 0.5736 | -0.01 | -0.00872 | 0.001282 | -0.005 | 0.000736 |
| 25 | 0.5833 | -0.123 | -0.12551 | 0.002507 | -0.07321 | 0.001463 |
| 26 | 0.59 | -0.21 | -0.20847 | 0.001528 | -0.123 | 0.000901 |

**Table A2**. *IAE* values of ERINMRIME on DDM

| Item | Measured data | | Current data simulated by ERINMRIME | | Power data simulated by ERINMRIME | |
| --- | --- | --- | --- | --- | --- | --- |
|  | $V(V)$ | $I(A)$ | $I_{S}(A)$ | $IAE_{I}(A)$ | $P_{S}(W)$ | $IAE_{P}(W)$ |
| 1 | -0.2057 | 0.764 | 0.763983 | 1.66E-05 | -0.15715 | 3.41E-06 |
| 2 | -0.1291 | 0.762 | 0.762604 | 0.000604 | -0.09845 | 7.8E-05 |
| 3 | -0.0588 | 0.7605 | 0.761338 | 0.000838 | -0.04477 | 4.93E-05 |
| 4 | 0.0057 | 0.7605 | 0.760174 | 0.000326 | 0.004333 | 1.86E-06 |
| 5 | 0.0646 | 0.76 | 0.759108 | 0.000892 | 0.049038 | 5.76E-05 |
| 6 | 0.1185 | 0.759 | 0.758121 | 0.000879 | 0.089837 | 0.000104 |
| 7 | 0.1678 | 0.757 | 0.757189 | 0.000189 | 0.127056 | 3.16E-05 |
| 8 | 0.2132 | 0.757 | 0.756244 | 0.000756 | 0.161231 | 0.000161 |
| 9 | 0.2545 | 0.7555 | 0.755177 | 0.000323 | 0.192193 | 8.21E-05 |
| 10 | 0.2924 | 0.754 | 0.753722 | 0.000278 | 0.220388 | 8.12E-05 |
| 11 | 0.3269 | 0.7505 | 0.751399 | 0.000899 | 0.245632 | 0.000294 |
| 12 | 0.3585 | 0.7465 | 0.747301 | 0.000801 | 0.267908 | 0.000287 |
| 13 | 0.3873 | 0.7385 | 0.740011 | 0.001511 | 0.286606 | 0.000585 |
| 14 | 0.4137 | 0.728 | 0.727247 | 0.000753 | 0.300862 | 0.000312 |
| 15 | 0.4373 | 0.7065 | 0.70685 | 0.00035 | 0.309106 | 0.000153 |
| 16 | 0.459 | 0.6755 | 0.675211 | 0.000289 | 0.309922 | 0.000133 |
| 17 | 0.4784 | 0.632 | 0.630761 | 0.001239 | 0.301756 | 0.000593 |
| 18 | 0.496 | 0.573 | 0.571995 | 0.001005 | 0.283709 | 0.000499 |
| 19 | 0.5119 | 0.499 | 0.499706 | 0.000706 | 0.2558 | 0.000361 |
| 20 | 0.5265 | 0.413 | 0.413734 | 0.000734 | 0.217831 | 0.000386 |
| 21 | 0.5398 | 0.3165 | 0.317546 | 0.001046 | 0.171411 | 0.000565 |
| 22 | 0.5521 | 0.212 | 0.212123 | 0.000123 | 0.117113 | 6.79E-05 |
| 23 | 0.5633 | 0.1035 | 0.102163 | 0.001337 | 0.057549 | 0.000753 |
| 24 | 0.5736 | -0.01 | -0.00879 | 0.001208 | -0.00504 | 0.000693 |
| 25 | 0.5833 | -0.123 | -0.12554 | 0.002543 | -0.07323 | 0.001484 |
| 26 | 0.59 | -0.21 | -0.20837 | 0.001628 | -0.12294 | 0.000961 |

**Table A3**. *IAE* values of ERINMRIME on TDM

| Item | Measured data | | Current data simulated by ERINMRIME | | Power data simulated by ERINMRIME | |
| --- | --- | --- | --- | --- | --- | --- |
|  | $V(V)$ | $I(A)$ | $I_{S}(A)$ | $IAE_{I}(A)$ | $P_{S}(W)$ | $IAE_{P}(W)$ |
| 1 | -0.2057 | 0.764 | 0.763986 | 1.4E-05 | -0.15715 | 2.88E-06 |
| 2 | -0.1291 | 0.762 | 0.762606 | 0.000606 | -0.09845 | 7.82E-05 |
| 3 | -0.0588 | 0.7605 | 0.761338 | 0.000838 | -0.04477 | 4.93E-05 |
| 4 | 0.0057 | 0.7605 | 0.760173 | 0.000327 | 0.004333 | 1.86E-06 |
| 5 | 0.0646 | 0.76 | 0.759106 | 0.000894 | 0.049038 | 5.77E-05 |
| 6 | 0.1185 | 0.759 | 0.758119 | 0.000881 | 0.089837 | 0.000104 |
| 7 | 0.1678 | 0.757 | 0.757186 | 0.000186 | 0.127056 | 3.12E-05 |
| 8 | 0.2132 | 0.757 | 0.756241 | 0.000759 | 0.161231 | 0.000162 |
| 9 | 0.2545 | 0.7555 | 0.755175 | 0.000325 | 0.192192 | 8.27E-05 |
| 10 | 0.2924 | 0.754 | 0.753721 | 0.000279 | 0.220388 | 8.16E-05 |
| 11 | 0.3269 | 0.7505 | 0.751399 | 0.000899 | 0.245632 | 0.000294 |
| 12 | 0.3585 | 0.7465 | 0.747303 | 0.000803 | 0.267908 | 0.000288 |
| 13 | 0.3873 | 0.7385 | 0.740014 | 0.001514 | 0.286607 | 0.000586 |
| 14 | 0.4137 | 0.728 | 0.727251 | 0.000749 | 0.300864 | 0.00031 |
| 15 | 0.4373 | 0.7065 | 0.706854 | 0.000354 | 0.309107 | 0.000155 |
| 16 | 0.459 | 0.6755 | 0.675212 | 0.000288 | 0.309922 | 0.000132 |
| 17 | 0.4784 | 0.632 | 0.63076 | 0.00124 | 0.301756 | 0.000593 |
| 18 | 0.496 | 0.573 | 0.571992 | 0.001008 | 0.283708 | 0.0005 |
| 19 | 0.5119 | 0.499 | 0.499703 | 0.000703 | 0.255798 | 0.00036 |
| 20 | 0.5265 | 0.413 | 0.413731 | 0.000731 | 0.217829 | 0.000385 |
| 21 | 0.5398 | 0.3165 | 0.317545 | 0.001045 | 0.171411 | 0.000564 |
| 22 | 0.5521 | 0.212 | 0.212124 | 0.000124 | 0.117114 | 6.86E-05 |
| 23 | 0.5633 | 0.1035 | 0.102166 | 0.001334 | 0.05755 | 0.000751 |
| 24 | 0.5736 | -0.01 | -0.00879 | 0.001211 | -0.00504 | 0.000694 |
| 25 | 0.5833 | -0.123 | -0.12554 | 0.002542 | -0.07323 | 0.001483 |
| 26 | 0.59 | -0.21 | -0.20837 | 0.001625 | -0.12294 | 0.000959 |

**Table A4**. *IAE* values of ERINMRIME on the PV module model

| Item | Measured data | | Current data simulated by ERINMRIME | | Power data simulated by ERINMRIME | |
| --- | --- | --- | --- | --- | --- | --- |
|  | $V(V)$ | $I(A)$ | $I_{S}(A)$ | $IAE_{I}(A)$ | $P_{S}(W)$ | $IAE_{P}(W)$ |
| 1 | 0.1248 | 1.0315 | 1.029119 | 0.002381 | 0.128434 | 0.000297 |
| 2 | 1.8093 | 1.03 | 1.027381 | 0.002619 | 1.858841 | 0.004738 |
| 3 | 3.3511 | 1.026 | 1.025742 | 0.000258 | 3.437363 | 0.000865 |
| 4 | 4.7622 | 1.022 | 1.024107 | 0.002107 | 4.877003 | 0.010035 |
| 5 | 6.0538 | 1.018 | 1.022292 | 0.004292 | 6.188751 | 0.025982 |
| 6 | 7.2364 | 1.0155 | 1.019931 | 0.004431 | 7.380627 | 0.032063 |
| 7 | 8.3189 | 1.014 | 1.016363 | 0.002363 | 8.455024 | 0.019659 |
| 8 | 9.3097 | 1.01 | 1.010496 | 0.000496 | 9.407417 | 0.00462 |
| 9 | 10.2163 | 1.0035 | 1.000629 | 0.002871 | 10.22273 | 0.02933 |
| 10 | 11.0449 | 0.988 | 0.984548 | 0.003452 | 10.87424 | 0.038122 |
| 11 | 11.8018 | 0.963 | 0.959522 | 0.003478 | 11.32408 | 0.04105 |
| 12 | 12.4929 | 0.9255 | 0.922839 | 0.002661 | 11.52893 | 0.033245 |
| 13 | 13.1231 | 0.8725 | 0.8726 | 9.97E-05 | 11.45121 | 0.001308 |
| 14 | 13.6983 | 0.8075 | 0.807274 | 0.000226 | 11.05829 | 0.003092 |
| 15 | 14.2221 | 0.7265 | 0.728336 | 0.001836 | 10.35847 | 0.026119 |
| 16 | 14.6995 | 0.6345 | 0.637138 | 0.002638 | 9.36561 | 0.038777 |
| 17 | 15.1346 | 0.5345 | 0.536213 | 0.001713 | 8.115371 | 0.025927 |
| 18 | 15.5311 | 0.4275 | 0.429511 | 0.002011 | 6.670784 | 0.031239 |
| 19 | 15.8929 | 0.3185 | 0.318775 | 0.000275 | 5.066252 | 0.004363 |
| 20 | 16.2229 | 0.2085 | 0.20739 | 0.00111 | 3.364461 | 0.018014 |
| 21 | 16.5241 | 0.101 | 0.096167 | 0.004833 | 1.589077 | 0.079857 |
| 22 | 16.7987 | -0.008 | -0.00833 | 0.000325 | -0.13985 | 0.005465 |
| 23 | 17.0499 | -0.111 | -0.11094 | 6.36E-05 | -1.89145 | 0.001084 |
| 24 | 17.2793 | -0.209 | -0.20925 | 0.000247 | -3.61565 | 0.004272 |
| 25 | 17.4885 | -0.303 | -0.30086 | 0.002136 | -5.26165 | 0.037363 |

**Table A5**. ERINMRIME extracts the unknown parameters of SDM and DDM of SM55 at $25^{\circ}C$ and different irradiance

| Parameters | Irradiance | | | | | |
| --- | --- | --- | --- | --- | --- | --- |
|  |  |  |  |  | |  |
| **SDM** | | | | |
| $I_{ph}(A)$ | 0.692013599 | 1.382843649 | 2.070895945 | 2.760379595 | | 3.450104093 |
| $I_{sd}(A)$ | 1.30918E-07 | 1.00416E-07 | 1.55592E-07 | 1.43952E-07 | | 1.71132E-07 |
| $R_{s}(\Omega)$ | 0.31242043 | 0.396667052 | 0.330466277 | 0.337590449 | | 0.329150526 |
| $R_{sh}(\Omega)$ | 438.0430174 | 427.0594356 | 450.1006533 | 459.9107422 | | 483.8656175 |
| $n$ | 1.370895784 | 1.351985918 | 1.387576289 | 1.38114552 | | 1.395742455 |
| *RMSE* | 5.20542E-04 | 7.07608E-04 | 8.23951E-04 | 6.68580E-04 | | 1.14622E-03 |
| **DDM** | | | | |
| $I_{ph}(A)$ | 0.692013614 | 1.382295703 | 2.070896543 | 2.760381699 | | 3.450103564 |
| $I_{sd1}(A)$ | 2.55351E-19 | 4.93671E-08 | 1.55514E-07 | 4.99045E-18 | | 1.71154E-07 |
| $I_{sd2}(A)$ | 0.31240574 | 0.448029906 | 0.330502513 | 0.337590235 | | 0.329147706 |
| $R_{s}(\Omega)$ | 438.0433683 | 475.6944206 | 450.0685447 | 459.878485 | | 483.9004709 |
| $R_{sh}(\Omega)$ | 3.070695891 | 1.297634579 | 1.387534233 | 3.903507832 | | 1.395752857 |
| $n_{1}$ | 1.30925E-07 | 1.00000E-04 | 5.10703E-19 | 1.43951E-07 | | 2.48246E-17 |
| $n_{2}$ | 1.370900441 | 4.00000E+00 | 3.682916109 | 1.381144509 | | 3.998613466 |
| *RMSE* | 5.20542E-04 | 5.99292E-04 | 8.23949E-04 | 6.68579E-04 | | 1.14621E-03 |

**Table A6**. ERINMRIME extracts the unknown parameters of SDM and DDM of ST40 at $25^{\circ}C$ and different irradiance

| Parameters | Irradiance | | | | | |
| --- | --- | --- | --- | --- | --- | --- |
|  | $200W/\text{m}^{2}$ | $400W/\text{m}^{2}$ | | $600W/\text{m}^{2}$ | $800W/\text{m}^{2}$ | $1000W/\text{m}^{2}$ |
| **SDM** | | |  |  |  |  |
| $I_{ph}(A)$ | 0.533137460 | 1.067544459 | | 1.604810169 | 2.138015836 | 2.675797482 |
| $I_{sd}(A)$ | 1.42961E-06 | 1.84897E-06 | | 1.44161E-06 | 1.15802E-06 | 1.52893E-06 |
| $R_{s}(\Omega)$ | 1.185743031 | 1.080558053 | | 1.112631389 | 1.125287682 | 1.113225081 |
| $R_{sh}(\Omega)$ | 344.9823559 | 362.5178573 | | 347.6835909 | 332.8786945 | 357.6378188 |
| $n$ | 1.747102679 | 1.778546543 | | 1.745101955 | 1.718664845 | 1.750336495 |
| *RMSE* | 4.77201E-04 | 6.30725E-04 | | 6.74036E-04 | 7.73906E-04 | 7.34099E-04 |
| **DDM** | | |  |  |  |  |
| $I_{ph}(A)$ | 0.533151949 | 1.067544227 | | 1.604809723 | 2.138014756 | 2.675799817 |
| $I_{sd1}(A)$ | 7.61503E-05 | 5.84532E-18 | | 2.46542E-16 | 3.74700E-18 | 1.52880E-06 |
| $I_{sd2}(A)$ | 1.40116573 | 1.08058031 | | 1.112613755 | 1.125286807 | 1.113225988 |
| $R_{s}(\Omega)$ | 360.4758767 | 362.5144969 | | 347.6946772 | 332.888942 | 357.5984327 |
| $R_{sh}(\Omega)$ | 4.00000E+00 | 3.83297414 | | 1.751580352 | 1.748626950 | 1.750326597 |
| $n_{1}$ | 6.1819E-07 | 1.84875E-06 | | 1.44187E-06 | 1.15810E-06 | 3.42198E-15 |
| $n_{2}$ | 1.644192251 | 1.77853025 | | 1.745123992 | 1.718673730 | 1.749859527 |
| *RMSE* | 4.54479E-04 | 6.30725E-04 | | 6.74036E-04 | 7.73905E-04 | 7.34099E-04 |

**Table A7**. ERINMRIME extracts the unknown parameters of SDM and DDM of KC200GT at $25^{\circ}C$ and different irradiance

| Parameters | Irradiance | | | | |
| --- | --- | --- | --- | --- | --- |
|  | $200W/\text{m}^{2}$ | $400W/\text{m}^{2}$ | $600W/\text{m}^{2}$ | $800W/\text{m}^{2}$ | $1000W/\text{m}^{2}$ |
| **SDM** |  |  |  |  |  |
| $I_{ph}(A)$ | 1.647729643 | 3.289799854 | 4.933942742 | 6.571536611 | 8.218326282 |
| $I_{sd}(A)$ | 2.20054E-10 | 2.52153E-10 | 5.49858E-09 | 6.97174E-10 | 7.85789E-10 |
| $R_{s}(\Omega)$ | 1.127463226 | 0.692114545 | 0.271977983 | 0.45928377 | 0.475736288 |
| $R_{sh}(\Omega)$ | 700.5537735 | 723.3957589 | 739.3794913 | 783.3994537 | 761.9136976 |
| $n$ | 1.00000E+00 | 1.00000E+00 | 1.114080164 | 1.041457208 | 1.056829959 |
| *RMSE* | 1.42329E-03 | 1.33644E-03 | 1.34898E-03 | 1.29842E-03 | 1.20608E-03 |
| **DDM** |  |  |  |  |  |
| $I_{ph}(A)$ | 1.64772968 | 3.289799965 | 4.933942856 | 6.571536705 | 8.218393581 |
| $I_{sd1}(A)$ | 5.66658E-17 | 2.52141E-10 | 5.49796E-09 | 6.97116E-10 | 6.4796E-10 |
| $I_{sd2}(A)$ | 1.127465357 | 0.692136488 | 0.271999101 | 0.459293611 | 0.486704836 |
| $R_{s}(\Omega)$ | 700.5531994 | 723.396067 | 739.3792375 | 783.398162 | 765.3212948 |
| $R_{sh}(\Omega)$ | 3.978470473 | 1.00000E+00 | 1.114077071 | 1.041455166 | 1.050179588 |
| $n_{1}$ | 2.20053E-10 | 6.88338E-19 | 2.8616E-17 | 2.9895E-16 | 4.62873E-08 |
| $n_{2}$ | 1.00000E+00 | 3.615354223 | 3.892018533 | 3.923951666 | 1.911461265 |
| *RMSE* | 1.42329E-03 | 1.33644E-03 | 1.34898E-03 | 1.29842E-03 | 1.20587E-03 |

**Table A8**. ERINMRIME extracts the parameters of SDM and DDM of SM55 at different temperatures and $1000W/\text{m}^{2}$ irradiance

|  | Temperatures | | | |
| --- | --- | --- | --- | --- |
|  | $25^{\circ}C$ | | $40^{\circ}C$ | $60^{\circ}C$ |
| **SDM** | |  |  |  |
| $I_{ph}(A)$ | 3.450102983 | | 3.469136761 | 3.494609268 |
| $I_{sd}(A)$ | 1.71167E-07 | | 1.14519E-06 | 6.90901E-06 |
| $R_{s}(\Omega)$ | 0.329144467 | | 0.313093386 | 0.318708555 |
| $R_{sh}(\Omega)$ | 483.9003559 | | 533.0975028 | 484.8511333 |
| $n$ | 1.395759332 | | 1.417846503 | 1.405134238 |
| *RMSE* | 1.14622E-03 | | 3.78881E-03 | 3.78039E-03 |
| **DDM** | |  |  |  |
| $I_{ph}(A)$ | 3.450103565 | | 3.469137515 | 3.494608469 |
| $I_{sd1}(A)$ | 1.54377E-17 | | 1.14511E-06 | 6.9095E-06 |
| $I_{sd2}(A)$ | 0.329147706 | | 0.313095932 | 0.318705726 |
| $R_{s}(\Omega)$ | 483.900462 | | 533.0692268 | 484.8839727 |
| $R_{sh}(\Omega)$ | 3.96110549 | | 1.417839762 | 1.405141754 |
| $n_{1}$ | 1.71154E-07 | | 7.43516E-17 | 2.62401E-17 |
| $n_{2}$ | 1.395752855 | | 3.998661936 | 3.775140763 |
| *RMSE* | 1.14621E-03 | | 3.78881E-03 | 3.78039E-03 |

**Table A9.** ERINMRIME extracts the parameters of SDM and DDM of ST40 at different temperatures and $1000W/\text{m}^{2}$ irradiance

|  | Temperatures | | | | |
| --- | --- | --- | --- | --- | --- |
|  | $25^{\circ}C$ | $40^{\circ}C$ | | $50^{\circ}C$ | $70^{\circ}C$ |
| **SDM** |  | |  |  |  |
| $I_{ph}(A)$ | 2.675801893 | 2.68091228 | | 2.691969638 | 2.692329441 |
| $I_{sd}(A)$ | 1.52854E-06 | 5.66654E-06 | | 1.86792E-05 | 8.75224E-05 |
| $R_{s}(\Omega)$ | 1.113240155 | 1.129288095 | | 1.149594989 | 1.125888131 |
| $R_{sh}(\Omega)$ | 357.5754376 | 364.107302 | | 295.0040037 | 367.755773 |
| $n$ | 1.750306013 | 1.722567646 | | 1.717564774 | 1.727320978 |
| *RMSE* | 7.34099E-04 | 1.32141E-03 | | 1.82326E-03 | 7.77718E-04 |
| **DDM** |  | |  |  |  |
| $I_{ph}(A)$ | 2.675799816 | 2.680911956 | | 2.690099012 | 2.692329477 |
| $I_{sd1}(A)$ | 3.39784E-17 | 2.22045E-19 | | 3.03801E-05 | 1.56752E-16 |
| $I_{sd2}(A)$ | 1.113225984 | 1.129297714 | | 1.258431803 | 1.125888707 |
| $R_{s}(\Omega)$ | 357.5984431 | 364.1097388 | | 349.7157656 | 367.7532301 |
| $R_{sh}(\Omega)$ | 3.997519097 | 3.998252732 | | 1.848290118 | 3.65341994 |
| $n_{1}$ | 1.5288E-06 | 5.6661E-06 | | 1.19486E-09 | 8.75219E-05 |
| $n_{2}$ | 1.750326604 | 1.722557645 | | 1.003152491 | 1.727319872 |
| *RMSE* | 7.34099E-04 | 1.32141E-03 | | 1.54594E-03 | 7.77718E-04 |

**Table A10**. ERINMRIME extracts the parameters of SDM and DDM of KC200GT at different temperatures and $1000W/\text{m}^{2}$ irradiance

|  | Temperatures | | | |
| --- | --- | --- | --- | --- |
|  | $25^{\circ}C$ | | $50^{\circ}C$ | $75^{\circ}C$ |
| **SDM** | |  |  |  |
| $I_{ph}(A)$ | 8.218325852 | | 8.296964016 | 8.380115497 |
| $I_{sd}(A)$ | 7.86012E-10 | | 7.02584E-08 | 1.27252E-06 |
| $R_{s}(\Omega)$ | 0.4757062 | | 0.368183709 | 0.350302892 |
| $R_{sh}(\Omega)$ | 761.9225361 | | 826.1998345 | 638.1907336 |
| $n$ | 1.056836623 | | 1.087161739 | 1.084594683 |
| *RMSE* | 1.20608E-03 | | 1.44677E-03 | 2.79408E-03 |
| **DDM** | |  |  |  |
| $I_{ph}(A)$ | 8.218394503 | | 8.296512139 | 8.376285999 |
| $I_{sd1}(A)$ | 4.47016E-08 | | 6.43423E-08 | 1.21609E-05 |
| $I_{sd2}(A)$ | 0.486797925 | | 0.370676937 | 0.360637824 |
| $R_{s}(\Omega)$ | 765.3225744 | | 891.3425139 | 1016.522372 |
| $R_{sh}(\Omega)$ | 1.901982916 | | 1.082365292 | 1.482742896 |
| $n_{1}$ | 6.465E-10 | | 6.50044E-05 | 2.89174E-07 |
| $n_{2}$ | 1.050096843 | | 4.00000E+00 | 1.000003862 |
| *RMSE* | 1.20587E-03 | | 1.43963E-03 | 2.05340E-03 |
